# Antimony-resistant PGPR mitigates Sb toxicity and accumulation in peppers by restructuring rhizosphere microorganisms

**DOI:** 10.3389/fmicb.2025.1658223

**Published:** 2025-10-01

**Authors:** Xiangquan Sheng, Jianzhong Zhu, Wenqian Li, Juan Wan, Kangbo Wu, Pan Yang, Renyang Duan, Zeliang Yang, Jing Bai, Yu Zheng

**Affiliations:** ^1^College of Agriculture and Biotechnology, Hunan University of Humanities, Science and Technology, Loudi, Hunan, China; ^2^Hunan Provincial Collaborative Innovation Center for Field Weeds Control, Hunan University of Humanities, Science and Technology, Loudi, Hunan, China

**Keywords:** plant growth-promoting rhizobacteria (PGPR), antimony, accumulation, rhizosphere, *Capsicum annuum* L., co-occurrence networks

## Abstract

Plant growth-promoting rhizobacteria (PGPR) provide a sustainable biofertilizer strategy for remediating heavy metal-contaminated soils by enhancing plant stress resistance through rhizosphere microbiota interactions. However, the mechanisms by which PGPR modulate rhizosphere communities and plant growth under antimony (Sb) stress remain poorly understood. This study investigated the effects of inoculating Sb-tolerant *Cupriavidus* sp. S-8-2 in pepper (*Capsicum annuum* L.) cultivated under varying levels of Sb contamination (0, 500, 1,000 mg/kg), employing a combination of metagenomic profiling and physicochemical analyses. Pot experiments demonstrated that inoculation significantly enhanced plant growth and nutrient acquisition while alleviating oxidative stress in Sb-stressed plants. Crucially, it reduced Sb translocation, resulting in a 54.75% decrease in shoot Sb content, along with a 33.33% increase in leaf biomass and a 38.98% increase in root biomass under 1,000 mg/kg Sb treatment. In parallel, rhizosphere properties such as total nitrogen (TN), total phosphorus (TP), and soil organic matter (SOM) were improved, as evidenced by an 81.35% increase in acid phosphatase activity under the same Sb concentration. Microbiota analysis revealed that inoculation enriched stress-responsive bacterial phyla, such as Proteobacteria and Actinobacteria, as well as key functional genera associated with Sb tolerance (e.g., *Ramlibacter*) and nutrient cycling (e.g., *Nitrospira*), despite a decrease in alpha-diversity. Co-occurrence networks analysis indicated that inoculation significantly enhance node connectivity and mean degree in rhizosphere bacterial networks, reflecting an increase in structural complexity, especially under severe Sb stress (1,000 mg/kg). These findings demonstrate that *Cupriavidus* sp. S-8-2 enhances plant resistance to Sb by restructuring the rhizobacterial community and improving soil health, with reducing Sb accumulation in edible parts, thereby highlighting its potential as a biofertilizer for safe crop production in Sb-contaminated soils. For the first time, our study explored the potential of Sb-tolerant PGPR to alleviate Sb stress in pepper plants cultivated in Sb-polluted soils.

## 1 Introduction

Soil is the fundamental foundation of agricultural resources, environmental quality, food security and remains vital for world sustainability. However, with the development of industrialization and urbanization, soils have become increasingly polluted by heavy metal(loid)s (HMs), which threaten food safety and human health. Sb, a toxic metalloid categorized as a priority pollutant by the U.S. Environmental Protection Agency (USEPA) and more recently by China in 2022, has been linked to potential carcinogenic effects and multi-organ toxicity through chronic exposure, primarily due to bioaccumulation within food chains (Zheng et al., 2023). In some countries, Sb concentrations in soils affected by mining activities have been reported to significantly exceed the maximum permissible limit of 36 mg/kg, which is established based on potential risks to human health as outlined by the World Health Organization (WHO). For instance, in China, elevated Sb levels in agricultural soils near mining areas have been documented, ranging from 101 to 5,045 mg/kg (Kong et al., 2024), surpassing the national average Sb concentration of 2.98 mg/kg in surface soils (Zhu et al., 2025). Similarly, the soil Sb concentration in an abandoned open-sky Sb mine in Djebel Hamimat, Algeria, in North Africa reached as high as 15,549 mg/kg (Zhao et al., 2023). The combination of Sb and sulfhydryl groups (-SH) within the human body can inhibit the activity of mercapto-iodoacetamide, interfere with the metabolism of proteins and carbohydrates, damage vital organs such as the liver and heart, affect the nervous system, and cause mucosal irritation (Zhao et al., 2023). In plants, Sb stress disrupts cellular homeostasis by interfering nutrient uptake, inhibiting root growth, and inducing oxidative damage via excessive production of reactive oxygen species (ROS) (Vidya et al., 2022). These phytotoxic effects not only reduce crop yield but also facilitate the transfer of Sb into edible plant tissues, thereby compromising food safety (Haider et al., 2024). For example, rice cultivated in Sb-polluted soils which can contain 4.90 mg/kg of Sb (Huang et al., 2025), posing a great threat to human health through food chain (Ye et al., 2018). Therefore, effective management and remediation strategies for Sb-contaminated soils are essential to ensure ecosystem sustainability and safeguard human health.

A variety of remediation strategies based on physicochemical methods have been implemented to rehabilitate contaminated agriculture soils; however, these approaches are frequently limited by high implementation costs, the potential for reduced soil fertility, and the risk of secondary pollution secondary pollution (Dutta et al., 2025). The development of biological alternative methods is essential for achieving optimal decontamination of HM-polluted soils at minimal cost while preserving or enhancing soil quality and fertility. Importantly, these methods must prevent the translocation of HMs from roots to edible plant tissues. In contrast to conventional approaches, bioremediation, particularly microbial-assisted strategies, has gained attention as a sustainable solution for mitigating HM toxicity and minimizing HM accumulation in crops.

PGPR have been recognized as key contributors to enhancing plant resilience under HM stress. This is achieved through multiple mechanisms, including the improvement of resource acquisition (e.g., nitrogen fixation, phosphorus solubilization, and essential mineral uptake), modulation of plant hormone levels, and stimulation of soil enzyme activities (Gupta et al., 2024). In the context of growing global emphasis on green sustainable agriculture, environmental protection, and food security, the application of PGPR represents a promising strategy for ensuring safe and enhanced production of food crops in HM-contaminated environments. The integration of PGPR into agricultural practices could provide an effective and environmentally friendly approach to mitigate the adverse effects of HM stress while promoting crop productivity and sustainability. Indeed, soil enzymes play a crucial role in the C (*β*-glucosidase and β-galactosidase), N (urease) and P (phosphatase) cycle (Daunoras et al., 2024), thereby improving soil functionality and promoting plant growth in this process. For instance, Abdelkrim et al. (2020) quantitatively demonstrated that *Lathyrus sativus*-PGPR significantly increased key soil enzyme activities such as acid phosphatase, alkaline phosphatase, and urease activities under Pb and Cd polluted sites. Similarly, inoculation with phosphate-solubilizing *Pseudomonas* sp. WS32 in wheat has been shown to increase plant growth and phosphorus uptake (Ou et al., 2022). Additionally, PGPR can also alleviate ROS-mediated oxidative stress in plants through the production of various antioxidant molecules in plants (Luo et al., 2024). A study demonstrated that inoculation with *Rhizobium* sp. RP5 increased the activity of antioxidant enzymes, including superoxide dismutase (SOD), catalase (CAT), and glutathione reductase (GSH) under stress conditions (Wani et al., 2008). Furthermore, HM-tolerant PGPR strains can mitigate HM toxicity by facilitating processes such as biotransformation, adsorption, precipitation, mineralization, and HM ion chelation. These mechanisms contribute to reducing HM bioavailability and uptake in plants (El-Meihy et al., 2019; Gupta et al., 2024). For instance, inoculation with *Bacillus* sp. MN3–4 has been shown to tolerate Pb and produce indole-3-acetic acid (IAA) and siderophores, which promote plant growth and enhance Pb accumulation in the hyperaccumulator *Alnus firma*; whereas two As-resistant PGPR bacterial strains (*Burkholderia cepacia* LAR-21 and LAR-25) have been shown to significantly reduce As levels in lentil seed tissues (Laha et al., 2024). Therefore, PGPR inoculation represents an effective strategy for restoring the quality and fertility of HM contaminated soils.

Research has also demonstrated that PGPR-mediated growth enhancement manifests through both physiological traits and metabolic responses. Chlorophyll, the primary pigment responsible for photosynthesis, plays a critical role in light-energy conversion and is essential for the synthesis of organic compounds in plants (Pavlović et al., 2014). Studies have shown that inoculation with *Pseudomonas aeruginosa* and *Burkholderia* spp. significantly increased total chlorophyll content in tomato plants, thereby enhancing photosynthetic activity-a key mechanism supporting plant growth and productivity in contaminated environments (Khanna et al., 2019b). Flavonoids, a major class of secondary metabolites in plants, are involved in critical signaling and defense functions during cellular development and stress responses (Patil et al., 2024). In chickpea plants inoculated with *Azospirillum brasilense* EMCC1454 elevated flavonoid levels were observed, which were associated with enhanced Cd tolerance and improved growth parameters. Conversely, uninoculated plants exhibited reduced flavonoid content (El-Ballat et al., 2023). Nevertheless, the coordinated regulation of these metabolites by PGPR under Sb stress remains largely unexplored, particularly in economically important crops such as pepper.

Emerging evidence highlights PGPR-induced shifts in rhizosphere microbiota as a pivotal mechanism for mitigating HM stress. Recent studies indicate that PGPR inoculation selectively enriches HM-resistant taxa, such as *Proteobacteria* and *Actinobacteria*, while suppressing pathogenic genera, such as *Fusarium* through niche competition under HM stress (Chaudhary et al., 2023). Soil microorganisms generally form complex networks through positive, negative, and neutral interactions, which play a critical role in shaping microbial community structures and, consequently, influence ecosystem functions. Coyte et al. (2015) proposed that the limitation of positive feedback loops and the reduction of ecological interactions are indicative of a greater resilience capacity within the community, enabling it to return to a stable state following environmental disturbances. Moreover, PGPR-mediated optimization of co-occurrence networks may enhance interaction intensity, leading to a more organized and efficient microbial community under stressful conditions (Kong et al., 2019). These PGPRs have been found to effectively bioremediate HM–contaminated soil by enhancing plant tolerance to HM stress, improving soil nutrient availability, modifying HM uptake pathways, and producing chemical compounds such as siderophores and chelating ions (Gupta et al., 2024). Despite extensive studies highlighting the beneficial effects of PGPR inoculation on plant growth and phytoremediation potential in HM-contaminated agricultural soils, including those polluted with As and Cd, relatively limited research has systematically investigated their efficacy under Sb contamination. Additionally, the precise mechanisms underlying PGPR modulation of the rhizosphere microbiome remain to be fully elucidated. Therefore, future research is essential to bridge these knowledge gaps and further explore the potential applications of PGPR in remediation strategies for Sb-contaminated environments.

In the preliminary phase of this study, *Cupriavidus* sp. S-8-2 was isolated from the rhizosphere of ferns grown in the Xikuangshan (XKS) mine, which is the world’s largest Sb mine located in Hunan Province, China (Zheng et al., 2023). This bacterium exhibited remarkable tolerance to various HMs, particularly Sb, and displayed multiple plant growth-promoting (PGP) activities under Sb stress (Zheng et al., 2023). Furthermore, this strain significantly reduced Sb accumulation and enhanced biomass in *Brassica napus* during seed germination under Sb stress (Zheng et al., 2023), thereby highlighting its substantial potential for promoting crop growth in Sb-contaminated agricultural soils.

Pepper (*Capsicum annuum* L.) is recognized as a globally important vegetable crop due to its nutritional and economic importance. China is the world’s largest producer of peppers, with the highest planting area and output worldwide. Both its production volume and economic revenue rank first among all vegetables in the country. However, due to its high market demand, pepper is commonly cultivated in soils contaminated with HMs, particularly Sb, in Hunan, China, especially in regions near mining sites. Unfortunately, plants, including peppers, are highly vulnerable to the toxic effects of Sb (Haider et al., 2024). A health risk assessment indicated that the hazard quotient (HQ) values of Sb in vegetables from the XKS region ranged from 1.61 to 3.33, surpassing the threshold value of 1, indicating a potential for serious health risks (Tang et al., 2022). Furthermore, Feng et al. (2013) estimated that the daily Sb intake among local residents in XKS was 554 μg, exceeding the established tolerant daily intake (TDI) of 360 μg. As a result, the accumulation of Sb in edible tissues of pepper may also poses increasingly serious challenges and present significant risks to human health (Zhou and Liu, 2024). Therefore, it is imperative to enhance Sb resistance in pepper plants while simultaneously reducing Sb accumulation and improving biomass and nutritional value for safe cultivation practices. Although many studies have highlighted that the application of PGPR serves as an eco-friendly and sustainable agricultural strategy, there is currently no research investigating the potential of HM-tolerant PGPR in mitigating Sb stress in pepper plants cultivated in Sb-polluted soils.

We hypothesized that *Cupriavidus* sp. S-8-2 would reduce Sb accumulation, promote plant growth, and enhance Sb stress tolerance in peppers through modulating rhizosphere microbiota and the strengthening of plant antioxidant defense mechanisms. The objectives of this study were as follows: (1) to investigate the effects of *Cupriavidus* sp. S-8-2 inoculation on plant growth, soil quality, and Sb uptake in pepper tissues; (2) to elucidate the mechanisms by which *Cupriavidus* sp. S-8-2 alleviates Sb stress in plants through biochemical responses within the plant-rhizosphere system; (3) to evaluate changes in the composition and function of the rhizosphere microbial community following inoculation with *Cupriavidus* sp. S-8-2 under varying Sb concentration. This study aims to provide a scientifically robust strategy for improving the remediation of agriculture soils contaminated with Sb while ensuring the sustainable development of agriculture and food safety.

## 2 Materials and methods

### 2.1 Experimental materials

Pepper (*Capsicum annuum* L.) seeds with uniform size and viability were obtained from the Agricultural Science Research Institute of Loudi City, Hunan Province, China. The seeds were surface-sterilized by immersion in a 2% (v/v) sodium hypochlorite (NaClO) solution for 30 min, followed by five rinses with sterile deionized water. After 25 days of soil germination under controlled conditions (14 h photoperiod, 25/20°C day/night temperature, and 80% relative humidity), morphologically homogeneous seedlings were selected and transplanted into experimental pots (20 cm diameter × 18 cm height) containing prepared growth substrate.

The Sb-tolerant PGPR strain *Cupriavidus* sp. S-8-2 was isolated from the rhizosphere of ferns collected from the XKS mine, according to our previously published protocol (Zheng et al., 2023). The bacterium was cultured in lysogeny broth (LB) medium (10 g/L tryptone, 5 g/L yeast extract, 10 g/L NaCl) at 30°C with shaking at 120 rpm for 48 h. Cells were harvested during mid-exponential growth phase (OD_600_ ≈ 1.0) by centrifugation at 5,000 g for 10 min, washed twice with sterile physiological saline (8.5 g/L NaCl), and resuspended to a final concentration of 1 × 10^9^ CFU/mL as determined by OD_600_ calibration.

Surface soil (0–20 cm depth) was sampled from uncontaminated agricultural fields at the Jiuer Experimental Station, located in Loudi City (27°44’N, 111°59′E), with the background Sb concentration of 1.96 mg/kg. The soil exhibited a yellow-brown color with the following physicochemical properties: organic matter content of 18.2 g/kg, cation exchange capacity of 15.3 cmol/kg, total phosphorus content of 1.97 g/kg, total nitrogen content of 3.22 g/kg, and pH of 5.41. Following air-drying, homogenization, and sieving through a 2 mm mesh to remove debris, soils were amended with potassium antimony tartrate (C_8_H_4_K_2_O_12_Sb_2_, analytical purity, Sigma) to achieve target Sb concentrations of 500 and 1,000 mg/kg Sb (dry weight basis), based on previously reported low-to-moderate contaminations levels in agricultural soils near Sb mining areas (Kong et al., 2024). The contaminated soils were then equilibrated for 28 days at 25 ± 1°C, with moisture maintained at 60% water-holding capacity, to facilitate Sb aging.

### 2.2 Pot experiment design and treatments

A pot experiment was carried out from April to July 2024 to evaluate the alleviation of Sb stress in pepper plants through inoculation with *Cupriavidus* sp. S-8-2. Morphologically uniform seedlings (25-day-old) were transplanted into pots (23.5 cm diameter × 29.0 cm height; 4 kg soil/pot). Six treatments were implemented: uninoculated control (0 mg/kg Sb; UCK), inoculated control (0 mg/kg Sb + *Cupriavidus* sp. S-8-2; ICK), uninoculated low Sb stress (500 mg/kg Sb; ULT), inoculated low Sb stress (500 mg/kg Sb + *Cupriavidus* sp. S-8-2; ILT), uninoculated high Sb stress (1,000 mg/kg Sb; UHT), and inoculated high Sb stress (1,000 mg/kg Sb + *Cupriavidus* sp. S-8-2; IHT). The experiment was conducted using a completely randomized block design, incorporating four biological replicates for each treatment group (4 biological replicates × 6 treatments = 24 pots in total). The bacterial suspension, containing 1 × 10^9^ CFU/mL, was prepared by resuspending pelletized cells in sterile distilled water following centrifugation (8,000 × g, 6 min) to remove residual growth media. It was then applied to the rhizosphere at 0, 30, and 45 days post-transplantation. Control treatments received equivalent volumes of sterile distilled water to ensure comparability and eliminate potential confounding effects associated with nutrient addition. The pots were maintained under natural photoperiod conditions, with daily irrigation to maintain 60–70% of the water holding capacity, at ambient temperature ranging from 19°C (night) to 28°C (day). Measured Sb concentrations in spiked soils showed 4.6% mean deviation from nominal values (<5.2% at 500–1000 mg/kg; Supplementary Figure S1).

### 2.3 Plant harvesting, samples collections, and Sb accumulation in each tissue of pepper plants

Plant samples were harvested at the fruit-bearing stage (120 days after post-sowing). Four biological replicates per treatment were randomly collected following a stratified random sampling protocol. Rhizosphere soil (approximately 200 g), adhering to roots within 1–2 mm, was carefully collected using gentle brushing and shaking for subsequent analysis. Roots were systematically separated, and subsamples were processed as follows: one portion was air-dried and sieved (0.074 mm) for the determination of pH, TN, TP, and SOM; a second portion was analyzed for total Sb concentration; a third portion was immediately placed in sterile cryovials, transported under refrigeration at 4°C, and stored at −80°C for enzyme activity assays and microbial community analysis.

Root systems were meticulously excavated while maintaining their structural integrity. Plant organs (roots, stems, leaves, and fruits) were separated and sequentially washed with tap water followed by deionized water to remove surface particulates. Morphometric parameters (total root length, stem basal diameter measured with digital caliper ±0.01 mm, and fresh biomass recorded via analytical balance ±0.01 g) were quantified immediately after processing. For the quantification of Sb concentration, tissues were freeze-dried at −50°C, ground into particles smaller than 0.5 mm in size, and subsequently digested using microwave-assisted digestion. The Sb content in each tissue was determined by inductively coupled plasma mass spectrometry (ICP-MS, model 7500c, Agilent Technologies, United States), following established methodology (Zheng et al., 2023).

### 2.4 Determination of chlorophyll and carotenoid content in leaves of pepper plants

Each fresh leaf sample (0.2 g fresh weight) was collected at harvest, with midribs carefully excised. The samples were subsequently homogenized in 20 mL of ice-cold 95% ethanol (HPLC grade, Sigma-Aldrich) using a mortar and pestle. Extraction was proceeded in amber glass vials at 4°C for 18 h until complete tissue depigmentation was achieved. The extracts were then adjusted to a final volume of 50 mL with 95% ethanol, followed by centrifugation at 8,000 × g for 10 min at 4°C. The concentrations of Chlorophyll a and chlorophyll b were determined spectrophotometrically (Shimadzu UV-1800) based on absorbance measurements at 665 nm and 649 nm, respectively, and calculated using Lichtenthaler’s equations (1987).

For carotenoids quantification, fresh leaf tissues (0.2 g) were thoroughly pulverized in liquid nitrogen and extracted with an acetone: petroleum ether (1:1 v/v) mixture under dim light conditions. Following vortexing for 2 min, the samples were centrifugated at 8,000 × g for 10 min at 4°C. The resulting supernatants were collected, pooled, and subsequently evaporated under N_2_ stream. The residues were then reconstituted in 5 mL of acetone, and absorbance was measured at 450 nm. The carotenoid content was determined according to the protocol outlined in the reference material (Zhang et al., 2020).

### 2.5 Determination of rhizosphere physicochemical properties and enzyme activities

Soil pH was determined in a 1:2 (w/v) soil-water suspension using a calibrated pH meter. TN content was quantified via semi-micro-Kjeldahl digestion and subsequently analyzed using a TOC-TN analyzer (Vario EL III, Elementar, Germany). SOM was assessed through potassium dichromate (K₂Cr₂O₇) wet oxidation followed by colorimetric determination. TP was quantified after HF-HClO₄ digestion using the molybdenum blue method.

Rhizosphere soil enzyme activities were analyzed post-harvest. Urease activity was determined by quantifying ammonia release following urea substrate incubation. Acid phosphatase activity was assayed by measuring p-nitrophenol (PNP) liberation from p-nitrophenyl phosphate disodium (pH 6.0, 115 mM) after 1 h incubation at 37°C. Saccharase activity was evaluated using sucrose as the substrate with 3,5-dinitrosalicylic acid reagent, with reducing sugars measured at 508 nm. All enzyme activities were quantified spectrophotometrically (UV-1800, Shimadzu), following the protocol provided.

### 2.6 Determination of root antioxidant enzymatic activities, MDA content, and total flavonoids in pepper plants

Fresh root tips were harvested and promptly snap-frozen in liquid nitrogen to halt metabolic activity, followed by storage at-80°C. Subsequently, samples (100 μg) were homogenized in ice-cold phosphate-buffered saline (PBS, pH 7.4) and centrifuged at 8,000 g for 20 min at 4°C. The resulting supernatants were filtered through double-layers cheesecloth to remove insoluble materials. Activities of SOD, POD, and CAT activities were quantified using commercial kits (Nanjing Jiancheng Bioengineering Institute, Nanjing, China) according to the manufacturer’s protocols. Enzyme activities were expressed as units per milligram of protein (U/mg protein), with protein concentrations determined using detection kits from the same supplier. Malondialdehyde (MDA) content, a widely recognized biomarker of membrane lipid peroxidation, was assessed using lipid peroxidation assay kits (Nanjing Jiancheng Bioengineering Institute, Nanjing, China) following the provided protocol.

For the determination of total flavonoids, 0.5 g dried root tissue was finely pulverized in liquid nitrogen. The resulting powder was extracted with 10 mL 80% methanol in a 50-mL polypropylene tube, followed by vortex mixing for 1 min and ultrasonic treatment at 45°C for 45 min. Subsequently, the extracts were filtered through Whatman No. 1 filter paper to remove insoluble residues. The total flavonoids content was quantified using the aluminum chloride colorimetric method as described by Hussain et al. (2023) (details in Supplementary Text S1).

### 2.7 Rhizosphere microbiome analysis

The rhizosphere microbiome was characterized through 16S rRNA gene sequencing. Genomic DNA was extracted from samples using the CTAB/SDS method (Andriyanto et al., 2022). The V3–V4 hypervariable region of the 16S rRNA gene, which offers superior taxonomic resolution across bacterial phyla while maintaining high sequencing accuracy, was selected for analysis (Klindworth et al., 2013). The region was amplified via PCR with a thermocycler under conditions specified by Li H. et al. (2020) and Li X. et al. (2020). PCR products were purified with the GeneJET™ Gel Extraction kit (Thermo Fisher Scientific, United States) to remove non-target DNA fragments. Sequencing libraries were prepared using the TruSeq^®^ DNA PCR-Free Sample Preparation kit (Thermo Fisher Scientific) in strict accordance with the manufacturer’s protocols. Rigorous quality control included: removal of PCR duplicates through unique molecular identifier filtering, excision of non-target fragments via GeneJET™ Gel Extraction (Thermo Fisher), and elimination of chimeric sequences during bioinformatic processing using DADA2 within QIIME2. Purified amplicons underwent library preparation with TruSeq^®^ DNA PCR-Free kits and were sequenced on Illumina HiSeq X Ten/NovaSeq 6,000 platforms (Thermo Fisher Scientific), yielding single-end reads of 400 bp or 600 bp. Full bioinformatic workflows including ASV clustering, alpha/beta diversity calculations, and PICRUSt2 functional predictions are detailed in Supplementary Text S2.

### 2.8 Data presentation and statistical analysis

IBM SPSS (v26.0.0, IBM Corp., United States) was employed for statistical analysis, while data visualization was conducted with Origin 9.1 (Origin Lab, United States). To assess the effects of inoculation treatments on soil properties, a one-way analysis of variance (ANOVA) coupled with Fisher’s least significant difference (LSD) *post hoc* test was performed at a 95% confidence interval (*α* = 0.05). The significance of differences between inoculated and non-inoculated groups was categorized as follows: *p* < 0.05 (*, moderate), *p* < 0.01 (**, high), and *p* < 0.001 (***, extreme). In graphical representations, distinct lowercase letters above bars indicate statistically significant differences (*p* < 0.05) derived from post hoc pairwise comparisons. Microbial co-occurrence networks were constructed using Spearman correlations (|r| > 0.6, *p* < 0.05) and visualized in Gephi. Redundancy analysis (RDA) and Mantel tests linked soil parameters to microbial composition. Structural Equation Model (SEM) analysis was conducted to explore the effects of soil chemical properties (pH, SOM, TP, and TN), enzyme activity and microbial community on plant Sb extraction efficiency using the “plspm” package in R. Detailed information is available in the Supplementary Text S3. All experiments were conducted with four biological replicates, and results are expressed as mean values ± standard error (SE).

## 3 Results

### 3.1 Effect of *Cupriavidus* sp. S-8-2 on pepper growth under Sb stress

Inoculation with *Cupriavidus* sp. S-8-2 significantly enhanced pepper growth across all treatments (Table 1). Root length and fresh weight, leaf fresh weight, and fresh fruit biomass all exhibited significant increases following inoculation, under both Sb-free and Sb-stressed environments. Specifically, root length increased by 6.86% (*p* < 0.05) in the absence of Sb stress, by 14.35% (*p* < 0.001) at 500 mg/kg Sb, and by 24.88% (*p* < 0.001) at 1000 mg/kg Sb. Root fresh weight increased by 29.10% (*p* < 0.001) without Sb stress, by 36.97% (*p* < 0.01) at 500 mg/kg Sb, and by 19.38% (*p* < 0.01) at 1000 mg/kg Sb. Leaf fresh weight increased by 33.33% (*p* < 0.01) under both Sb concentrations. Furthermore, inoculation significantly enhanced fresh fruit biomass by 18.48% (*p* < 0.05) at the highest Sb level, suggesting that *Cupriavidus* sp. S-8-2 contributes to improved reproductive development under Sb stress.

**Table 1:** Effects of *Cupriavidus* sp. strain S-8-2 inoculation on pepper plant growth and biomass allocation under varying Sb concentrations after 120 days.

Treatment	Root length (mm)	Root weight (g·FW)	Stem diameter (mm)	Fruit weight (g·FW)	Leaf weight (g·FW)	Total plant weight (g·FW)
UCK	25.96 (±0.13)^cd^	54.40 (±0.31)^b^	7.04 (±0.95)^b^	14.87 (±1.15)^a^	0.33 (±0.01)^b^	52.76 (±1.38)^d^
ICK	27.74 (±0.61)^c^	70.23 (±0.94)^a^	9.47 (±0.33)^a^	15.32 (±0.71)^a^	0.40 (±0.02)^a^	88.89 (±2.00)^a^
ULT	35.19 (±2.44)^b^	49.50 (±1.38)^b^	7.45 (±0.69)^ab^	11.71 (±0.48)^bc^	0.27 (±0.01)^c^	63.91 (±2.02)^c^
ILT	40.24 (±0.70)^a^	67.80 (±2.78)^a^	8.72 (±0.67)^ab^	12.50 (±0.73)^c^	0.38 (±0.02)^a^	81.92 (±1.09)^b^
UHT	23.93 (±0.36)^d^	39.12 (±2.03)^c^	7.87 (±0.77)^ab^	11.99 (±0.32)^ab^	0.24 (±0.01)^c^	55.02 (±1.54)^d^
IHT	34.88 (±0.68)^b^	54.37 (±1.21)^b^	8.91 (±0.37)^ab^	14.10 (±0.39)^bc^	0.32 (±0.02)^b^	68.30 (±1.65)^c^
*F*-value (treatment)	32.927	49.920	1.977	5.076	14.817	77.156
*p*	<0.0001	<0.0001	NS	<0.01	<0.0001	<0.0001

UCK, ICK, ULT, ILT, UHT, and IHT, respectively, represented six treatments: uninoculated control (0 mg/kg Sb; UCK), inoculated control (0 mg/kg Sb + Cupriavidus sp. S-8-2; ICK), uninoculated low Sb stress (500 mg/kg Sb; ULH), inoculated low Sb stress (500 mg/kg Sb + Cupriavidus sp. S-8-2; ILH), uninoculated high Sb stress (1,000 mg/kg Sb; UHT), and inoculated high Sb stress (1,000 mg/kg Sb + Cupriavidus sp. S-8-2; IHT). Data represent means ± SE (*n* = 4). Different lowercase letters (a, b, c, etc.) denote significant differences between inoculated and non-inoculated treatments within Sb concentrations. Differences with *p* < 0.05 were considered statistically significant (LSD test).

### 3.2 Effect of *Cupriavidus* sp. S-8-2 on Sb accumulation in plant tissues under Sb stress

**Figure 1:**
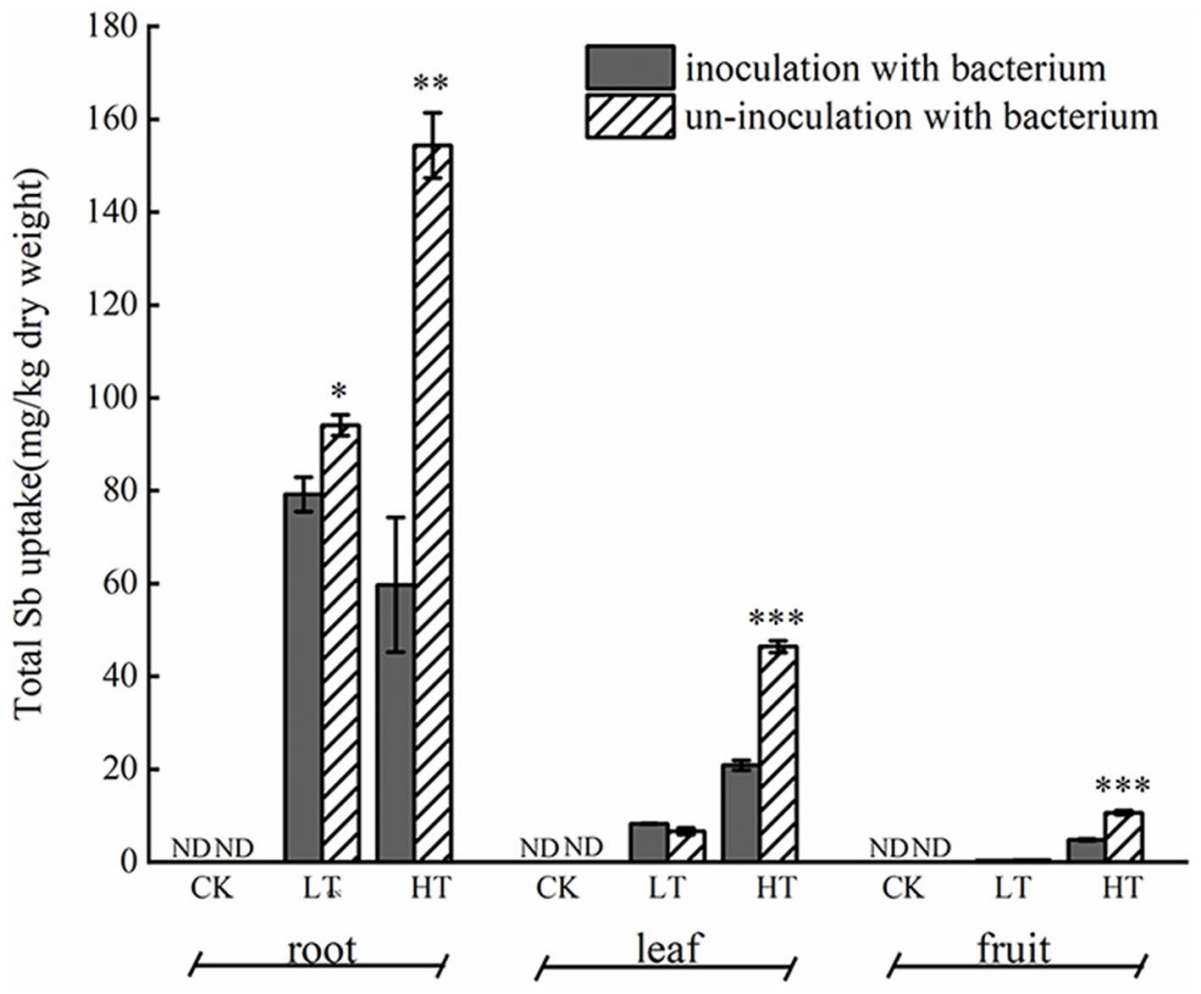
Effect of *Cupriavidus* sp. S-8-2 inoculation on Sb accumulation in roots, leaves, and fruit of pepper plants grown under different Sb concentrations after 120 days. The results are shown as the mean ± SE (*n* = 4) in each treatment replicate. Asterisks denote significant differences between inoculated and uninoculated treatments within the same Sb level (*p < 0.05, **p < 0.01, ***p < 0.001; one-way ANOVA). UCK (uninoculated control, 0 mg/kg Sb), ICK (Inoculated Control: 0 mg/kg Sb + *Cupriavidus* sp. S-8-2), ULT (uninoculated low Sb: 500 mg/kg Sb), ILT (inoculated low Sb: 500 mg/kg Sb + *Cupriavidus* sp. S-8-2), UHT (uninoculated high Sb: 1000 mg/kg Sb), IHT (inoculated High Sb: 1000 mg/kg Sb + *Cupriavidus* sp. S-8-2). ND, not detected.

Under Sb stress, the accumulation of Sb in roots, stems, and leaves was observed in both inoculated and non-inoculated pepper plants (Figure 1). In non-inoculated plants, the Sb content followed the order: roots > leaves > fruits, with the highest total Sb concentration recorded under Sb-only treatment conditions. Under 500 mg/kg Sb stress, no significant difference in Sb content was observed between inoculated and non-inoculated plants in leaf tissues. However, inoculation with *Cupriavidus* sp. S-8-2 significantly reduced Sb accumulation in fruit tissues (*p* < 0.01). Notably, under 1,000 mg/kg Sb stress, compared to non-inoculated treatment, the Sb content decreased by 61.31% in roots (*p* < 0.01), 55.02% in leaves (*p* < 0.001), and 54.75% in fruits (*p* < 0.001).

### 3.3 Effect of *Cupriavidus* sp. S-8-2 on chlorophyll and carotenoids content in pepper leaves under Sb stress

**Figure 2:**
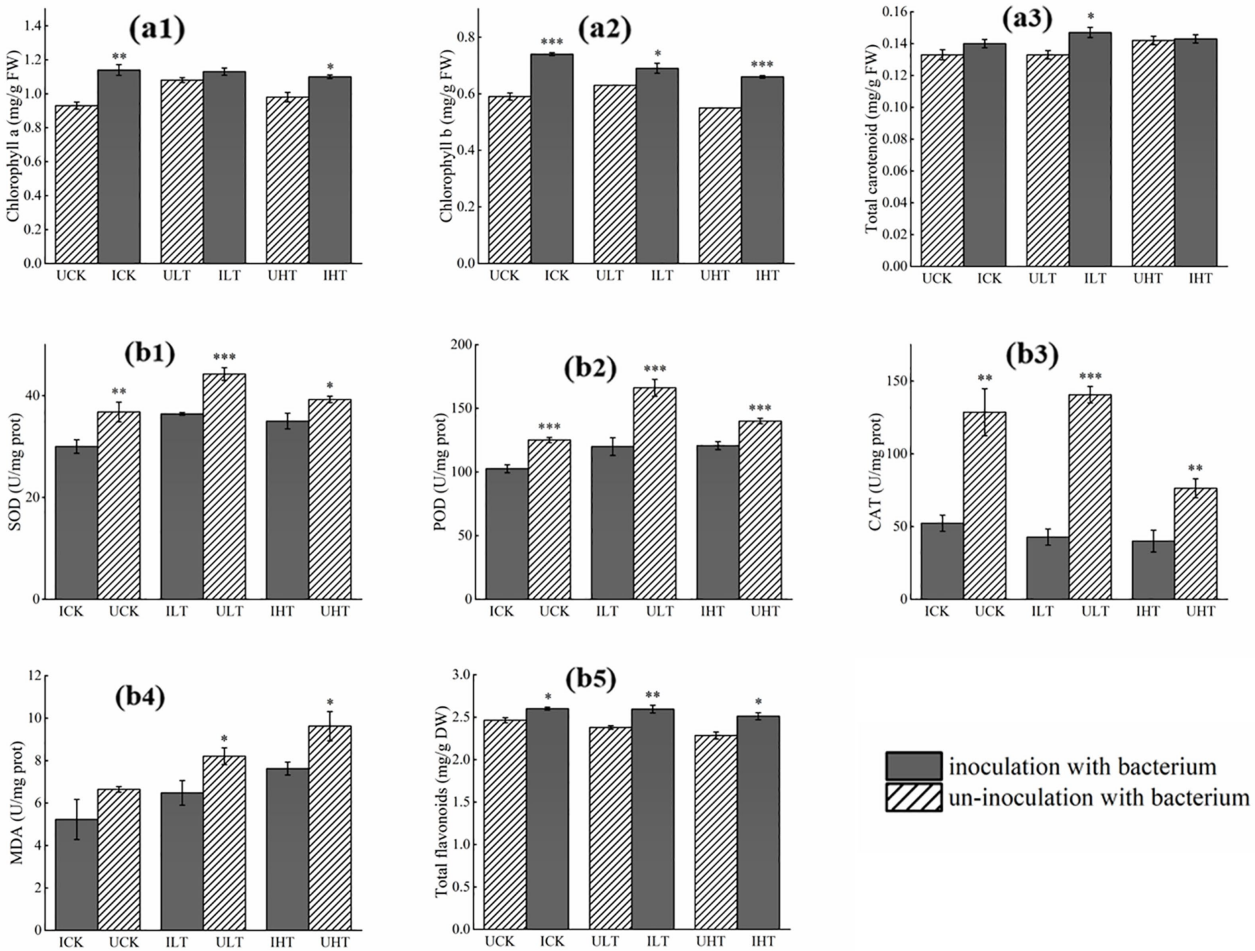
Biochemical responses in pepper plants inoculated with *Cupriavidus* sp. S-8-2 under varying Sb concentrations after 120 days. **(A)** Leaf pigments: Chlorophyll a (a1), chlorophyll b (a2), and carotenoids content (a3); **(B)** Root antioxidant parameters: Superoxide dismutase (SOD, b1), peroxidase (POD, b2), catalase (CAT, b3), malondialdehyde (MDA, b4), and total flavonoids (b5). Data represent means ± SE (*n* = 4 treatment replicates). Asterisks indicate significant differences between inoculated and uninoculated treatments within the same Sb level (* *p* < 0.05, ** *p* < 0.01, *** *p* < 0.001; one-way ANOVA). UCK (uninoculated control, 0 mg/kg Sb), ICK (inoculated control: 0 mg/kg Sb + *Cupriavidus* sp. S-8-2), ULT (uninoculated low Sb: 500 mg/kg Sb), ILT (inoculated low Sb: 500 mg/kg Sb + *Cupriavidus* sp. S-8-2), UHT (uninoculated high Sb: 1000 mg/kg Sb), IHT (inoculated high Sb: 1000 mg/kg Sb + *Cupriavidus* sp. S-8-2).

Inoculation with *Cupriavidus* sp. S-8-2 significantly increased chlorophyll a content in pepper leaves by 22.58% (*p* < 0.01) under Sb-free conditions and 12.25% (*p* < 0.05) under 1,000 mg/kg Sb stress (Figure 2a1). Chlorophyll b content also showed marked improvements across all Sb treatments, with increases of 25.42% (*p* < 0.001) at 0 mg/kg Sb, 11.11% (*p* < 0.05) at 500 mg/kg Sb, and 20.00% (*p* < 0.001) at 1000 mg/kg Sb compared to non-inoculated treatments (Figure 2a2). Carotenoid levels remained stable at 0.133 ~ 0.147 mg/g. FW across treatments except under 500 mg/kg Sb stress, where inoculated plants exhibited a 10.52% increase relative to non-inoculated treatment (*p* < 0.05) (Figure 2a3).

### 3.4 Effect of *Cupriavidus* sp. S-8-2 on antioxidant enzyme activity, MDA content, and total flavonoids in pepper root under Sb stress

*Cupriavidus* sp. S-8-2 inoculation significantly altered root antioxidant enzyme activities relative to non-inoculated controls (Figure 2B). Specifically, SOD activity showed a significant decrease of 45.86% in the inoculated controls (*p* < 0.01) and a reduction of 21.50% under low Sb stress conditions (*p* < 0.001). In contrast, the decrease in SOD activity was not statistically significant under high Sb stress conditions (12.11%) (Figure 2b1). POD activity was reduced by 21.94% (*p* < 0.001), 38.48% (*p* < 0.001), and 16.67% (*p* < 0.001) under 0, 500, and 1,000 mg/kg Sb treatments, respectively (Figure 2b2). Similarly, CAT activity decreased by 1.45-fold (*p* < 0.01), 2.29-fold (*p* < 0.001), and 0.91-fold (*p* < 0.01) in corresponding treatments (Figure 2b3). MDA content, indicating lipid peroxidation, was consistently lower in inoculated roots, decreasing by 26.69% (*p* < 0.05) at 500 mg/kg and 27.52% (*p* < 0.05) at 1000 mg/kg Sb (Figure 2b4). Total flavonoids, contributing to stress tolerance, was elevated its contents by 9.24% (*p* < 0.01) and 8.77% (*p* < 0.05) at 500 mg and 1,000 mg Sb/kg, respectively (Figure 2b5). These results suggest that inoculation with *Cupriavidus* sp. S-8-2 mitigates oxidative damage through coordinated modulation of antioxidant defenses in Sb-stressed pepper roots.

### 3.5 Effect of *Cupriavidus* sp. S-8-2 on rhizosphere physicochemical properties and enzyme activities under Sb stress

The inoculation treatment significantly improved rhizosphere physicochemical properties under Sb stress (Table 2). SOM increased by 6.30, 4.50, and 8.40 g/kg under Sb concentrations of 0, 500, and 1,000 mg/kg, respectively (*p* < 0.05). TP levels increased by 26.42, 35.42, and 36.54% under the same Sb levels (*p* < 0.05). Acid phosphatase activity markedly decreased under Sb stress in the absence of inoculation, but increased by 48.33–81.35% when inoculated (*p* < 0.05). Urease activity was significantly enhanced by inoculation, with notably higher levels observed across all tested Sb concentrations, showing a 2.03-fold increase even under highest Sb concentration (1,000 mg/kg). CAT activity exhibited the most pronounced improvement following inoculation under non-Sb stress conditions. Although sucrase activity showed relatively weaker response to inoculation, increases were still evident under Sb stress compared to the uninoculated control treatments.

**Table 2:** Effects of *Cupriavidus* sp. strain S-8-2 inoculation on rhizosphere physicochemical properties and enzyme activities under varying Sb concentrations after 120 days.

Treatment	TN (g/kg)	TP (g/kg)	SOM (g/kg)	pH	Soil urease (μgNH_4_^+^-N /g·h)	Soil sucrase (mg glucose /g·h)	Soil catalase (mg H_2_O_2_ /g·h)	Soil Acid phosphatase (μg phenol /g·h)
UCK	0.42 (±0.02)^bcd^	0.53 (±0.05)^b^	37.00 (±1.30)^b^	4.99 (±0.09)^bc^	10.11 (±0.59)^c^	0.69 (±0.02)^b^	0.46 (±0.02)^b^	53.32 (±2.62)^d^
ICK	0.54 (±0.04)^a^	0.67 (±0.02)^a^	43.30 (±1.52)^a^	5.13 (±0.02)^a^	29.23 (±1.29)^a^	0.93 (±0.03)^a^	1.29 (±0.15)^a^	79.09 (±2.86)^a^
ULT	0.37 (±0.05)^cd^	0.48 (±0.05)^b^	32.73 (±0.76)^c^	4.96 (±0.01)^bc^	7.38 (±0.62)^d^	0.56 (±0.03)^c^	0.67 (±0.03)^b^	47.83 (±1.22)^d^
ILT	0.43 (±0.03)^bc^	0.65 (±0.01)^a^	37.23 (±1.52)^b^	5.06 (±0.01)^ab^	14.90 (±0.15)^b^	0.71 (±0.03)^b^	1.31 (±0.15)^a^	72.37 (±2.42)^b^
UHT	0.32 (±0.02)^d^	0.52 (±0.04)^b^	26.87 (±0.69)^d^	5.13 (±0.01)^a^	6.75 (±0.79)^d^	0.67 (±0.04)^b^	0.45 (±0.03)^b^	35.66 (±1.32)^e^
IHT	0.50 (±0.01)^ab^	0.71 (±0.02)^a^	35.27 (±1.47)^bc^	4.88 (±0.02)^c^	13.70 (±0.84)^b^	0.83 (±0.05)^a^	1.22 (±0.09)^a^	64.67 (±1.92)^c^
F-value (treatment)	6.634	7.594	18.605	6.288	109.310	14.622	18.501	56.758
*p*	0.004	0.002	<0.0001	0.0043	<0.0001	<0.0001	<0.0001	<0.0001

UCK, ICK, ULT, ILT, UHT, and IHT, respectively, represented six treatments: uninoculated control (0 mg/kg Sb; UCK), inoculated control (0 mg/kg Sb + Cupriavidus sp. S-8-2; ICK), uninoculated low Sb stress (500 mg/kg Sb; ULH), inoculated low Sb stress (500 mg/kg Sb + Cupriavidus sp. S-8-2; ILH), uninoculated high Sb stress (1,000 mg/kg Sb; UHT), and inoculated high Sb stress (1,000 mg/kg Sb + Cupriavidus sp. S-8-2; IHT). Data represent means ± SE (*n* = 4). Different lowercase letters (a, b, c, etc.) denote significant differences between inoculated and non-inoculated treatments within Sb concentrations. Differences with *p* < 0.05 were considered statistically significant (LSD test).

### 3.6 Effect of *Cupriavidus* sp. S-8-2 on rhizosphere microbial community structure and functional metabolism under Sb stress

The observed growth promotion and biochemical alterations underscored the role of *Cupriavidus* sp. S-8-2 in rhizosphere remodeling under Sb stress. To elucidate the mechanisms underlying these phenotypic enhancements, we performed Illumina sequencing of the rhizosphere microbiota. After stringent quality control, 1,158,364 high-quality sequences were clustered into 10,280–10,524 OTUs at a 97% similarity threshold (Supplementary Table S1), revealing 878 conserved OTUs across all treatments and 1,493–2,063 group-specific OTUs (Supplementary Figure S2A). Under 1,000 mg/kg Sb stress, inoculated treatments showed significantly higher OTU richness from 1771 to 1914 compared to the non-inoculated treatment (Supplementary Figure S2A). Alpha diversity exhibited distinct response to different treatments. Inoculated treatments significantly influenced all alpha-diversity indices (Shannon, Chao1, Simpson, Good’s coverage, Pielou’s evenness; *p* < 0.05), confirming microbial communities restructuring (Supplementary Figure S2B). The Chao1 index increased by 19.87% in the inoculated control under Sb-free conditions. Under low Sb stress (500 mg/kg), the Chao1 decreased by 6.16% following inoculation. Under high Sb stress (1,000 mg/kg), inoculation resulted in a 10.2% reduction in the Chao1 index. Notably, inoculation consistently reduced Shannon indices across all Sb stress levels relative to uninoculated counterparts.

Beta diversity analysis via PCoA demonstrated clear stratification among treatments under 1,000 mg/kg Sb stress (Supplementary Figure S3A), revealing three distinct groupings: (1) Sb-free groups (0 mg/kg with or without *Cupriavidu*s sp. S-8-2) formed a cohesive cluster; (2) moderate Sb stress (500 mg/kg, with or without *Cupriavidu*s sp. S-8-2) exhibited transitional grouping; and (3) high Sb treatments (1,000 mg/kg, with or without *Cupriavidu*s sp. S-8-2) showed unique segregation between each other. These results suggest threshold-dependent restructuring of microbial communities under varying Sb stress levels. The outcomes of ANOSIM also revealed inoculated communities clustered distinctly from non-inoculated counterparts (R = 1, *p* = 0.001, Supplementary Figure S3B).

**Figure 3:**
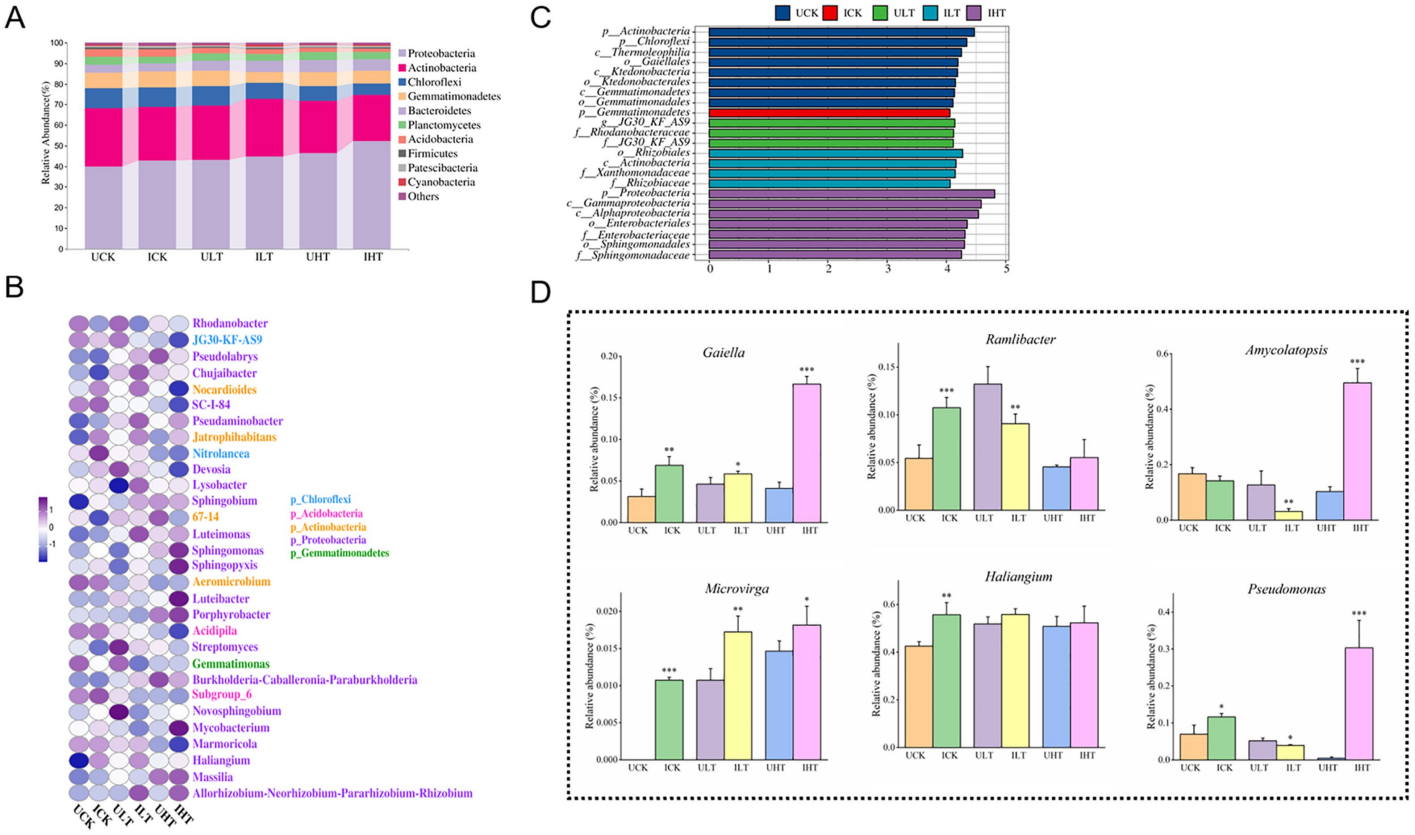
Rhizosphere microbial community structure of pepper plants inoculated with *Cupriavidus* sp. S-8-2 under varying Sb concentrations after 120 days. **(A)** Heatmap of relative abundance for the top 10 bacterial phyla; **(B)** Heatmap of relative abundance for the top 30 genera; **(C)** LEfSe analysis identifying taxa with significant differential abundance across treatments (LDA score > 4.0); **(D)** Relative abundance of selected significantly altered genera (one-way ANOVA, *p* < 0.05). Data represent means ± SE (*n* = 4 treatment replicates). Asterisks indicate significant differences between inoculated and uninoculated treatments within the same Sb level (*p < 0.05, **p < 0.01, ***p < 0.001; one-way ANOVA). UCK (uninoculated control, 0 mg/kg Sb), ICK (inoculated control: 0 mg/kg Sb + *Cupriavidus* sp. S-8-2), ULT (uninoculated low Sb: 500 mg/kg Sb), ILT (inoculated low Sb: 500 mg/kg Sb + *Cupriavidus* sp. S-8-2), UHT (uninoculated high Sb: 1000 mg/kg Sb), IHT (inoculated high Sb: 1000 mg/kg Sb + *Cupriavidus* sp. S-8-2).

The shifts in microbial community composition were visualized using heatmap analysis of the top 10 phyla and 30 genera across Sb concentration gradients (Figures 3A,B). Seven dominant phyla with relative abundances exceeding 1% were consistently identified (Figure 3A; Supplementary Table S2). The abundance of Proteobacteria generally increased with elevated Sb concentrations, peaking at 52.10% under 1,000 mg/kg Sb stress in inoculated treatments. In contrast, Acidobacteria exhibited a significant decline upon Sb exposure, with further suppression observed following inoculation compared to non-inoculated controls. Chloroflexi displayed Sb-dependent depletion, showing a 41.2% greater reduction in inoculated compared relative to non-inoculated ones across all Sb levels. Low-abundance phyla (<0.1%) displayed distinct ecological niches responses to Sb levels and inoculation (Supplementary Table S2). For instance, Chlamydia was detected exclusively in non-inoculated treatments and was undetectable in inoculated soils exposed to Sb concentrations exceeding 500 mg/kg. Elusimicrobia demonstrated Sb-sensitive colonization, being detected exclusively in Sb-free soils and inoculated soils treated with 500 mg/kg Sb. Notably, Cyanophyta showed an increased abundance with rising Sb concentration and exhibited a 1.9-fold higher abundance in inoculated treatments compared to non-inoculated ones under 1,000 mg/kg Sb stress. These findings underscore the distinct responses of microbial taxa to Sb exposure and emphasize the impact of *Cupriavidus* sp. S-8-2 inoculation on the structure of microbial community in the pepper rhizosphere.

The heatmap analysis of the Top 30 genera within the rhizosphere microbial community also revealed compositional differences between inoculated and non-inoculated treatments (Figure 3B; Supplementary Table S3). Dominant genera, defined as those with relative abundances exceeding 1%, included *Rhodanobacter*, *JG30-KF-AS9*, *Pseudolabrys*, *Chujaibacter*, *Nocardioides*, and *Jatrophihabitans*. Inoculated treatments exhibited reduced relative abundances of *Rhodanobacter* and *JG30-KF-AS9* compared to non-inoculated controls. Conversely, the relative abundances of *Pseudolabrys*, *Jatrophihabitans*, and *Saccharimonadales* were elevated in the inoculated treatments. The relative abundance of *Lysobacter* was significantly greater in inoculated treatments under low Sb stress conditions (0 and 500 mg/kg Sb*; p* < 0.05). *Luteimonas* and the *Allorhizobium-Neorhizobium-Pararhizobium-Rhizobium* group also showed increased abundance under 500 mg/kg Sb stress in inoculated treatments (*p* < 0.05). These genera continued to exhibit elevated abundances under 1,000 mg/kg Sb stress in inoculated treatments (*p* < 0.05). The abundance of *Nocardioides* increased significantly in inoculated treatments under 500 mg/kg Sb stress (*p* < 0.05). In contrast, *Conexibacter* decreased at 0 and 500 mg/kg Sb but increased at 1000 mg/kg in inoculated treatments (*p* < 0.05). *Flavobacterium* was detected only under Sb stress and showed higher abundance in inoculated treatments. Some rare genera with abundances below 0.1%, such as *Dyella*, *Gaiella*, and *Amycolatopsis*, exhibited significantly increased abundance in inoculated treatments under Sb stress (*p* < 0.05, Figure 3D).

The LEfSe analysis (LDA score > 4.0) identified distinct microbial biomarkers across treatments and Sb stress conditions (Figure 3C). Under Sb-free conditions, non-inoculated treatments were characterized by phylum-level biomarkers Actinobacteria and Chloroflexi. Conversely, inoculated treatments under the same conditions showed specific enrichment of Gemmatimonadetes at the phylum level. At a moderate Sb stress level (500 mg/kg), the genus *JG30-KF-AS9* was identified as a biomarker in non-inoculated treatments, whereas inoculated treatments were distinguished by the family *Rhizobiaceae*. Under elevated Sb stress (1,000 mg/kg Sb), two family-level biomarkers, *Enterobacteriaceae* and *Sphingomonadaceae* were observed in inoculated treatments. Notably, no significant biomarkers were detected in non-inoculated treatments exposed to 1,000 mg/kg Sb stress.

The functional prediction of rhizosphere microbial communities was performed using PICRUSt2 across KEGG metabolic hierarchies (Supplementary Figure S4). Inoculation with *Cupriavidus* sp. S-8-2 significantly upregulated multiple metabolic pathways associated with detoxification and stress resistance. Notably, under conditions of 1,000 mg/kg Sb, a marked enhancement was observed in pathways related to ABC transporter, including cofactor, prosthetic group, electron carrier degradation, and fatty acid and lipid biosynthesis. Interestingly, the activity of the tricarboxylic acid (TCA) cycle remained consistently higher in inoculated treatments compared to non-inoculated treatments across all tested Sb concentrations. Furthermore, analysis revealed a substantial upregulation of antibiotic resistance pathways in the inoculated treatments, particularly under 1,000 mg/kg Sb stress conditions. Concurrently, significant activation of secondary metabolite biosynthesis pathways was observed in the inoculated treatments.

**Figure 4:**
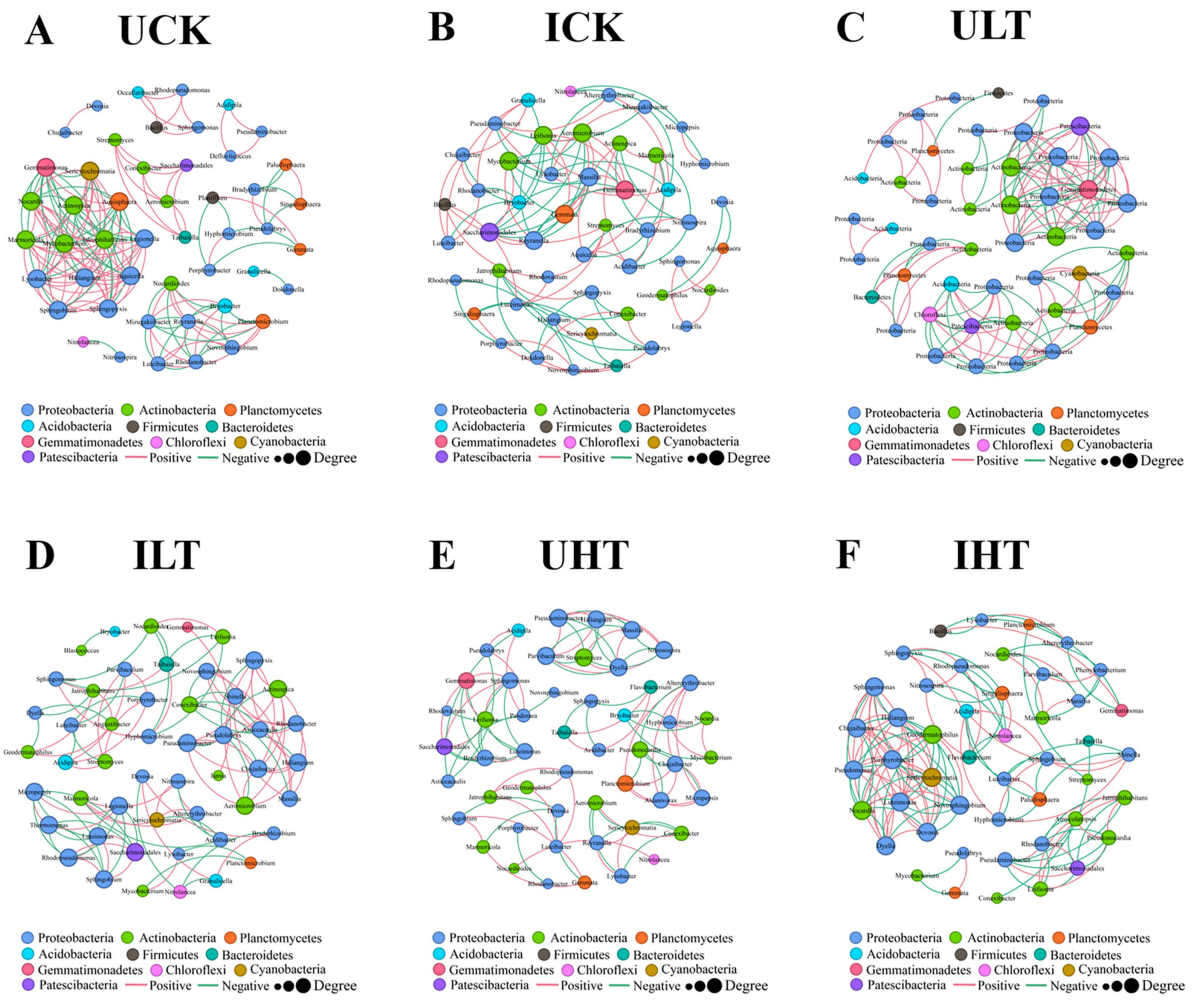
Rhizosphere bacterial co-occurrence networks across experimental treatments. Networks were constructed at the operational taxonomic unit (OTU) level using significant correlations (|r| > 0.6, *p* < 0.05). Node size scales with degree centrality (number of connections), reflecting ecological influence. Edges represent microbial interactions: red (positive correlations), green (negative correlations). UCK (uninoculated control, 0 mg/kg Sb), ICK (inoculated control: 0 mg/kg Sb + *Cupriavidus* sp. S-8-2), ULT (uninoculated low Sb: 500 mg/kg Sb), ILT (inoculated low Sb: 500 mg/kg Sb + *Cupriavidus* sp. S-8-2), UHT (uninoculated high Sb: 1000 mg/kg Sb), IHT (inoculated high Sb: 1000 mg/kg Sb + *Cupriavidus* sp. S-8-2).

The interspecific interactions were analyzed using SparCC-based co-occurrence networks (|r| > 0.60, *p* < 0.05). The network topology exhibited distinct treatment-specific variations (Figure 4; Table 3). The non-inoculated treatments under severe Sb stress (1,000 mg/kg) exhibited the least connected network structure, whereas the inoculated treatments under the same stress level demonstrated significantly higher connectivity. Notably, the average node degree was markedly increased in inoculated treatments under 1,000 mg/kg Sb stress compared to their non-inoculated counterparts. Additionally, a higher proportion of negative correlations was detected in inoculated treatments under 0 and 500 mg/kg Sb stress conditions. The analysis identified specific members of Actinobacteria, Proteobacteria, Acidobacteria, and Planctomycetes as occupying key topological positions, suggesting their roles as keystone species (Figure 4). Proteobacteria consistently displayed the highest level of network connectivity across all treatments, engaging in both positive and negative correlations. Under inoculation treatments at 1000 mg/kg Sb, significant antagonistic interactions were observed between Proteobacteria and Actinobacteria. Furthermore, inoculation treatments exhibited significantly enhanced intra-phylum connectivity within Proteobacteria compared to non-inoculation treatments. Inoculation notably enhanced intra-phylum connectivity within Proteobacteria, with this effect becoming more pronounced as Sb concentrations increased. Actinobacteria exhibited strong correlations with Planctomycetes in inoculated samples, forming a dense subnetwork that was most evident under high Sb stress. In the absence of Sb stress, *Bacillus* (phylum Firmicutes) acted as a central hub genus, showing correlations with PGPR-associated genera such as *Rhodanobacter* and *Luteibacter*; however, this specific regulatory network structure collapsed under moderate Sb stress (500 mg/kg). Under severe Sb stress condition (1,000 mg/kg), genera such as *Sericytochromatia* (phylum Cyanobacteria) and *Acidipila* (phylum Acidobacteria) partially compensated for the topological collapse by assuming hub-like functions. In contrast, non-inoculation treatments lacked such compensatory hubs, resulting in a marked reduction in overall network connectivity compared to their inoculated counterparts. Among the inoculated treatments under Sb stress, *Rhodanobacter* and *Luteimonas* emerged as core genera within two separate subnetworks, likely due to their divergent metabolic patterns; notably, *Luteimonas* displayed correlation patterns that were largely opposite to those of *Rhodanobacter*.

**Table 3:** Topological properties of co-occurrence networks for rhizosphere soils under different inoculation and Sb exposure treatments.

Topological index	UCK	ICK	ULT	ILT	UHT	IHT
Nodes	48	49	47	50	50	47
Edges	153	97	142	104	94	138
Positive count	95	48	80	58	49	69
Negative count	58	49	62	46	45	69
Density	0.1356	0.0824	0.1313	0.0848	0.0767	0.1276
Average degree	6.375	3.9591	6.0425	4.16	3.76	5.8723
Modularity	0.6033	0.8493	0.7141	0.8622	0.8671	0.7167
Negative vs. positive (%)	0.6105	1.0208	0.7750	0.7931	0.9183	1

UCK, ICK, ULT, ILT, UHT, and IHT, respectively, represented six treatments: uninoculated control (0 mg/kg Sb; UCK), inoculated control (0 mg/kg Sb + Cupriavidus sp. S-8-2; ICK), uninoculated low Sb stress (500 mg/kg Sb; ULH), inoculated low Sb stress (500 mg/kg Sb + Cupriavidus sp. S-8-2; ILH), uninoculated high Sb stress (1,000 mg/kg Sb; UHT), and inoculated high Sb stress (1,000 mg/kg Sb + Cupriavidus sp. S-8-2; IHT).

### 3.7 Correlation analysis of key soil factors and rhizosphere microbial communities under Sb stress

**Figure 5:**
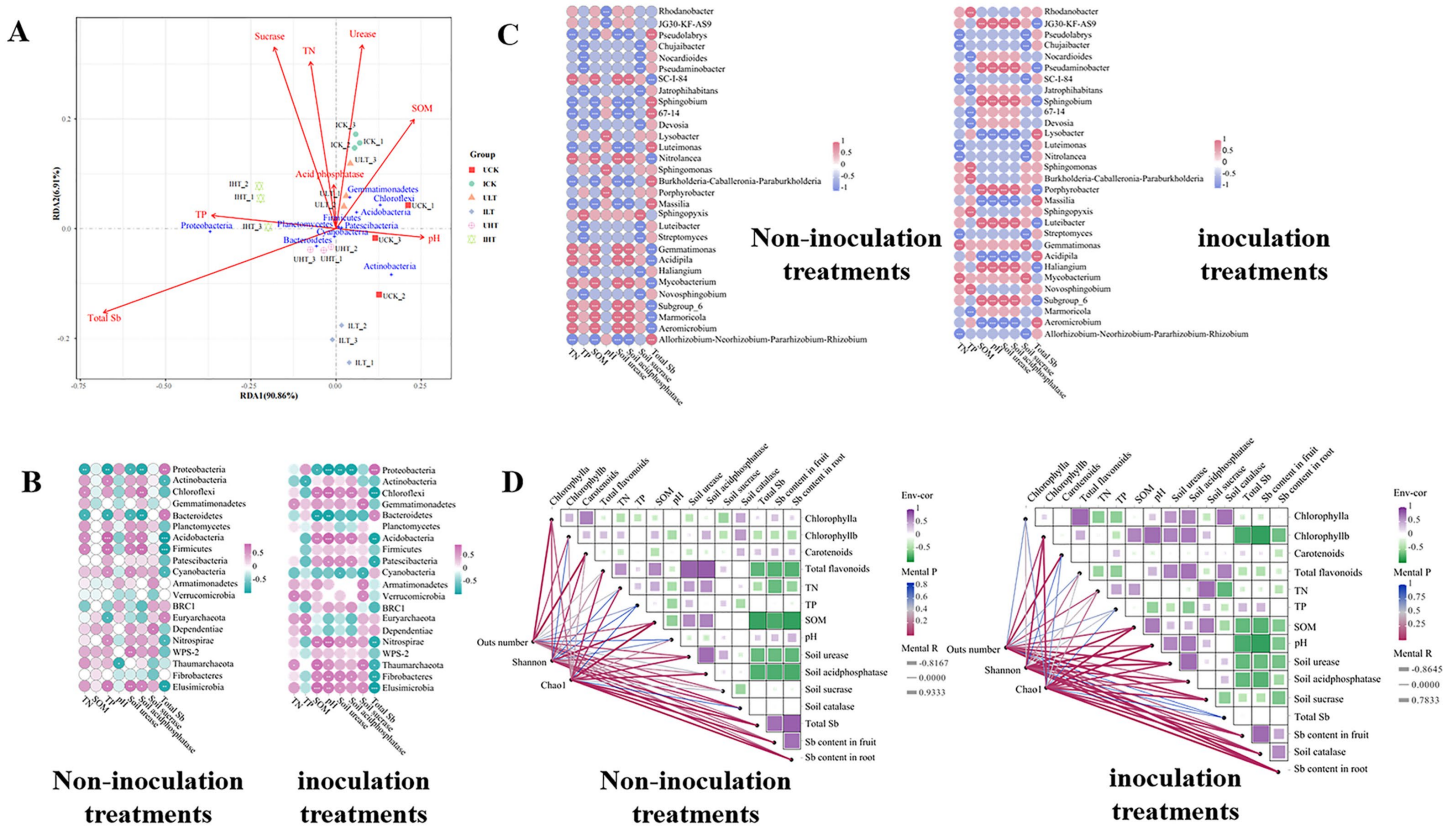
Drivers of rhizosphere bacterial community composition across treatments. **(A)** Redundancy analysis (RDA) of the top 10 most abundant bacterial phyla constrained by soil physicochemical properties. Arrows indicate environmental variables. Heatmaps of Pearson correlations between soil parameters and **(B)** top 20 phyla or **(C)** top 30 genera. Color gradients denote association strength (magenta/red: positive; blue: negative; white:|r| < 0.6 and *p* < 0.05). Significance: *p < 0.05, **p < 0.01, ***p < 0.001. **(D)** Mantel network linking bacterial community dissimilarity (Bray-Curtis) with edaphic parameter distances (Euclidean). Edge color intensity reflects Mantel r significance (999 permutations; red: *p* < 0.05). UCK (uninoculated control, 0 mg/kg Sb), ICK (inoculated control: 0 mg/kg Sb + *Cupriavidus* sp. S-8-2), ULT (uninoculated low Sb: 500 mg/kg Sb), ILT (inoculated low Sb: 500 mg/kg Sb + *Cupriavidus* sp. S-8-2), UHT (uninoculated high Sb: 1000 mg/kg Sb), IHT (inoculated high Sb: 1000 mg/kg Sb + *Cupriavidus* sp. S-8-2).

Redundancy analysis (RDA) was employed to characterize the environmental drivers influencing the phyla-level structure of the microbial community, explaining 97.77% of the total variance (Figure 5A). Among the analyzed factors, rhizosphere enzyme sucrase activity was identified as the most significant chemical factor affecting the distribution of rhizosphere bacterial communities across all treatments (*R*^2^ = 0.20, Figure 5A). Soi Sb concentration exhibited a significantly negative correlation with microbial community structure (*p* < 0.01). Urease activity, TN, and SOM were also recognized as key drivers shaping community structure. Chloroflexi, Acidobacteria, and Gemmatimonadetes correlated positively with urease and SOM, while Proteobacteria and Planctomycetes associated with sucrase, TN, and TP (*p* < 0.05). In contrast, sucrase activity, TN, and TP showed positively associated with the abundances of Proteobacteria and Planctomycetes (*p* < 0.05). At 1000 mg/kg Sb, the correlations between microbial communities and acid phosphatase, sucrase activity, SOM, and TN were significantly enhanced in inoculated treatments compared to non-inoculated treatments.

Pearson correlation analysis revealed strong associations among rhizosphere enzyme activities, physicochemical properties, and microbial abundance at both the phylum and genus levels across different treatments (Figures 5B,C). Total Sb exhibited significant negative effects on most bacterial phyla, especially Chloroflexi, Firmicutes, Nitrospirae, and Elusimicrobia, and multiple genera such as *SC-I-84*, *Nitrolancea*, *Subgroup_6*, *Acidibacter*, *Aeromicrobium*, and *Marmoricola*. In inoculated treatments, Chloroflexi (phylum) and *JG30-KF-AS9* (genus) exhibited significant negative correlations with total Sb (*p* < 0.01), while Firmicutes showed more variable trends. Total Sb was positively correlated with several phyla, including Proteobacteria, Bacteroidetes, and Euryarchaeota, as well as specific genera such as *Pseudolabrys*, *Sphingomonas*, *Massilia*, *Luteimonas*, and *Burkholderia-Caballeronia-Paraburkholderia*. Soil nutrients (TP and TN) exerted limited influence on microbial phyla regardless of treatment conditions. However, SOM and enzyme activities (urease, sucrase, and acid phosphatase) were positively influence specific phyla (Chloroflexi, Nitrospirae, and Fibrobacteres) and genera (*SC-I-84*, *JG30-KF-AS9*, *Aeromicrobium*, and *Marmoricola*), but negatively impacted other genera such as *Massilia*.

Mantel correlation analysis was performed to investigate the potential associations between various environmental factors and rhizosphere bacterial communities across different treatments (Figure 5D). Root Sb content exhibited a strong negative correlation with bacterial community structure following inoculation with *Cupriavidus* sp. S-8-2 (|r| > 0.6, *p* < 0.01), as reflected by in alpha-diversity metrics, including Observed OTUs, Chao1, and Shannon indices. In contrast, under non-inoculated conditions, inverse relationships were observed, suggesting that root Sb accumulation may disrupt the integrity and stability of the bacterial community. Moreover, SOM Additionally, SOM showed significant positive correlations with the Chao1 and Shannon indices in inoculated systems. Enzyme activity and soil pH were positively correlated with bacterial diversity under inoculated conditions (|r| > 0.6, *p* < 0.05). However, no significant correlations were detected between soil nutrients, including TP and TN, and microbial community indices across all treatments. Carotenoids content and chlorophyll levels were correlated with bacterial communities exclusively under non-inoculated conditions (|r| > 0.6, *p* < 0.05). while total flavonoids showed a negative correlation with tissue Sb content only under inoculated conditions. Furthermore, chlorophyll a was positively correlated with SOM, urease activity, and acid phosphatase (|r| > 0.6, *p* < 0.05) in inoculated treatments, while it exhibited a negative correlation with tissue Sb content under the same treatments.

### 3.8 Biotic and abiotic factors affecting pepper growth under Sb stress

**Figure 6:**
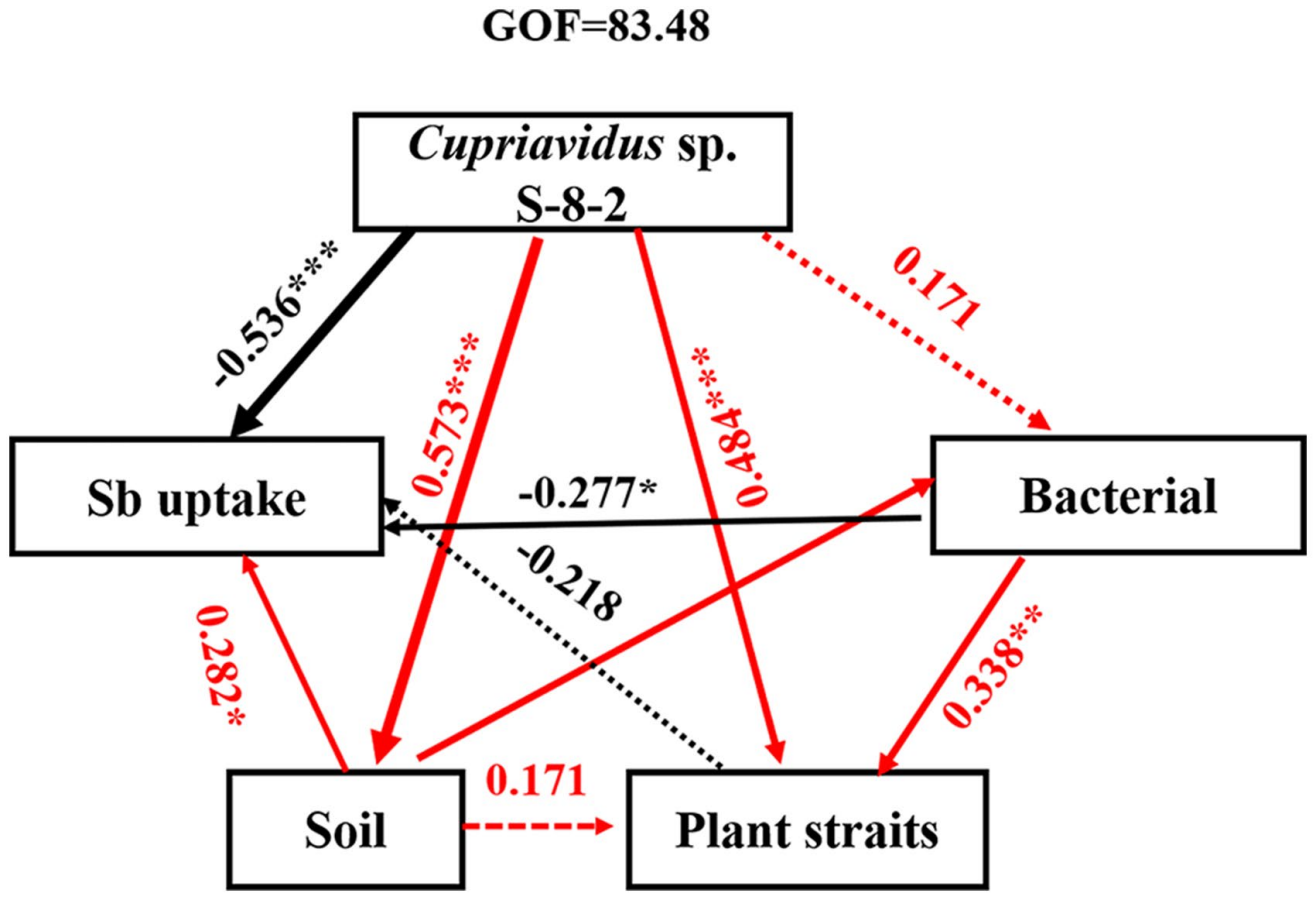
Structural equation model analysis of the contribution of soil physicochemical properties and microbial community to Sb accumulation of pepper plants inoculated with *Cupriavidus* sp. S-8-2 under varying Sb concentrations. GOF is the goodness-of-fit index. Dashed and solid lines denote insignificant and significant impacts, respectively (*p* < 0.05). Wider arrows represent higher path coefficients, while red and black lines indicate negative and positive effects, respectively. Coefficients of determination (*R*^2^) and path coefficients were calculated after 1,000 bootstraps. Significance: *p < 0.05, **p < 0.01, ***p < 0.001. Standardized impacts (indirect plus direct influences) were determined using the partial least squares-path models.

The SEM results revealed that inoculation with *Cupriavidus* sp. S-8-2 exerted both direct and indirect influence on the structure and diversity of the microbial community, which in turn affected microbial functions, plant growth, and Sb uptake through alterations in soil nutrient profiles (Figure 6). Among these factors, the correlation coefficient between *Cupriavidus* sp. S-8-2 inoculation and plant traits, including fresh weight, root height, and chlorophyll content was determined to be 0.484. In contrast, a significant negative correlation was observed between *Cupriavidus* sp. S-8-2 inoculation and Sb uptake, with a correlation coefficient of −0.536. Notably, the correlation coefficient between *Cupriavidus* sp. S-8-2 inoculation and bacteria abundance and diversity was 0.171, which was not statistically significant. This lack of significance may be attributed to the fact that inoculation with *Cupriavidus* sp. S-8-2 primarily alters microbial community structure and functions rather than directly influencing bacterial abundance or diversity. These findings collectively highlight the complex interactions between PGPR inoculation, plant performance, and nutrient dynamics in the soil environment under Sb stress.

## 4 Discussion

Building upon our previous identification of *Cupriavidus* sp. S-8-2 as a novel Sb-resistant PGPR capable of indole-3-acetic acid production, phosphate solubilization, and ACC deaminase activity under Sb stress, this strain was shown to significantly enhance morphological parameters and stress-responsive mechanisms during Sb-exposed rapeseed (*Brassica napus*) germination (Zheng et al., 2023). Although these findings highlight potential applications of the strain in Sb-contaminated agricultural bioremediation, its broader biotechnological applicability, particularly regarding Sb partitioning between soil matrices and edible tissues, as well as its functional impact on rhizosphere microbial communities in vegetable production systems remains largely unexplored. To address these knowledge gaps, we conducted a thorough evaluation of the responses of pepper (*Capsicum annuum* L.) to inoculation with strain S-8-2 under Sb stress using an integrated systems biology approach. This included the analysis of physiological parameters, tissue-specific Sb accumulations patterns, and rhizosphere microbial community profiling based on 16S rRNA gene sequencing. Furthermore, concurrent assessment of rhizosphere physicochemical properties and key enzymatic activities provided deeper insights into the strain’s potential in microecological engineering. Clarifying the mechanisms underlying Sb detoxification mediated by this PGPR may offer critical insights into ensuring safe crop production in Sb-contaminated agricultural environments, thereby contributing to sustainable agricultural practices.

### 4.1 Inoculation with *Cupriavidus* sp. S-8-2 mitigates Sb-induced growth inhibition in pepper plants

Inoculation with *Cupriavidus* sp. S-8-2 significantly alleviated the inhibitory effects of Sb on the growth of pepper plants, highlighting its potential application as a bioremediation agent in soils contaminated with low-to-medium level of Sb (Sb ≤ 1,000 mg/kg). In this study, inoculation with strain S-8-2 markedly enhanced root length, biomass, and chlorophyll content in pepper plants exposed to Sb stress. These enhancements can be attributed to the plant growth-promoting (PGP) properties of strain S-8-2, including its capability to regulate phytohormone levels, such as IAA, as our previously reported (Zheng et al., 2023). Such hormonal regulation may facilitate root elongation and enhance overall plant development. Notably, inoculated plants exhibited higher chlorophyll content compared to non-inoculated controls, particularly under exposure to 1,000 mg/kg Sb (Figure 2). Research has shown that HMs can accumulate in chlorophyll molecules, triggering their degradation, a process is often associated with ROS-mediated interference (Karimi et al., 2025). Conversely, several studies have shown that inoculation with Sb-tolerant PGPR strains can increase chlorophyll content in plants grown under HM stress (Aziz et al., 2025). Additionally, carotenoids are known to function as a photo-protective pigment. A reduction in carotenoids content has been previously observed in various plants under HM stress due to ROS-mediated inhibition of carotenogenesis (Espinola et al., 2025). However, PGPR inoculation enhanced carotenoid levels in *Sesbania sesban* under stress conditions (Zainab et al., 2021), a result consistent with our observations. The elevated chlorophyll and carotenoid content can be attributed to the ability of PGPR to upregulate carotenogenic genes, maintain chloroplast structural integrity, enhance the efficiency of photosynthetic photon capture, and suppress ROS-dependent pigment degradation (Bhardwaj et al., 2024; Li X. et al., 2022; Li Z. et al., 2022).

Exposure to HM stress increases ROS accumulation in plants, which activates the antioxidant defense system and enhances antioxidant enzyme activity (Mansoor et al., 2023). Among these antioxidant enzymes, SOD, POD, and CAT play crucial roles in ROS scavenging (Song et al., 2025). Studies have demonstrated that synergistic action of SOD and CAT effectively reduces ROS levels, thereby creating favorable conditions for photosynthesis (Moustaka et al., 2025; Rahim et al., 2025). In the present study, inoculation with *Cupriavidus* sp. S-8-2 significantly attenuated the activities of POD, CAT, POD, and SOD in peppers across all tested Sb concentration (Figure 2). These attenuations may be attributed to *Cupriavidus* sp. S-8-2 substantially reducing Sb accumulation in root tissues, thereby mitigating Sb-induced toxicity to pepper roots. This reduction in Sb uptake was associated with decreased ROS levels and modulation of antioxidant enzyme activities. Similar observations were reported by Park et al. (2017), who found that inoculation of soybean plants with PGPR *Bacillus aryabhattai* resulted in decreased CAT and POD activities. Likewise, Cd-tolerant PGPR strains (*Burkholderia gladioli* and *Pseudomonas aeruginosa*) have been shown to reduce Cd uptake and subsequently lower antioxidant enzyme levels in tomato plants (Khanna et al., 2019a). Additionally, MDA, a key biomarker of lipid peroxidation and oxidative damage, was significantly reduced in pepper plants inoculated with *Cupriavidus* sp. S-8-2 compared to non-inoculated controls. Consistent with this, treatment with *Bacillus subtilis* strain IU31 resulted in decreased MDA levels in rice plants exposed to As stress (Ullah et al., 2024). Furthermore, previous studies have demonstrated that flavonoids can suppress ROS biosynthesis and reduce MDA content, thereby alleviating plant stress under adverse conditions. Supporting this, Wu et al. (2025) reported that inoculation with *Pseudomonas koreensis* in Cd-stressed rice significantly increased total flavonoids content, which was accompanied by enhanced antioxidant capacity, chlorophyll content, and biomass accumulation. Taken together, these observations are consistent with the present findings, indicating that inoculation with Sb-tolerant PGPR effectively alleviates Sb-induced stress, reduces oxidative damage, and enhances the survival and growth of pepper plants under high Sb conditions.

### 4.2 Inoculation with *Cupriavidus* sp. S-8-2 modulates rhizosphere physicochemical properties and enzyme activities in the pepper plants under Sb stress

Plant roots critically regulate the dynamics at the soil-root interface by modifying rhizosphere physicochemical properties. In the present study, inoculation with *Cupriavidus* sp. S-8-2 under high Sb stress (1,000 mg/kg) significantly decreased rhizosphere pH from 5.13 to 4.88 (Table 2). This acidification likely resulted from enhanced secretion of low-molecular-weight organic acids (e.g., malate, citrate, and oxalate), which facilitate Sb immobilization through precipitation mechanisms under elevated Sb stress (Zheng et al., 2023). Furthermore, HMs can form stable complexes with SOM, including humic and fulvic acids, as evidenced by the observed relationship between SOM and pH in the inoculated treatments under 1,000 mg/kg Sb stress. Conversely, under moderate Sb stress (500 mg/kg), inoculated treatments exhibited increased rhizosphere pH compared to non-inoculated controls. This observation suggests that organic acid secretion may be limited under lower stress conditions, thereby exerting minimal influence on the pH of the soil solution. Instead, alternative mechanisms that result in a net increase in rhizosphere pH may predominate under such conditions (Kangi, 2024). Further investigation into the underlying processes governing these differential pH responses could enhance our understanding of the adaptive strategies employed by plant–microbe systems under varying levels of Sb stress.

Concurrently, HM contamination triggered alterations in soil chemical processes while potentially suppressing biological activities linked to nutrient cycling. This shift appears to align with changes in specific soil physicochemical properties, such as decreased soil enzyme activities. These observations are consistent with the findings obtained under elevated Sb exposure. However, inoculation with *Cupriavidus* sp. S-8-2 significantly increased soil TN, TP, and SOM contents compared to the uninoculated controls (Table 2). These results suggest that PGPR inoculation enhances soil quality through augmenting nutrient pools. Our observations are in agreement with Ju et al. (2019), who reported that PGPR inoculation significantly improved soil properties and fertility in copper-contaminated system by elevating tissue nutrient (N, P, and K) content and promoting plant growth. Soil enzymes may indirectly influence carbon dynamics through nutrient leaching or microbial activity modulation, while also directly participating in the conversion of organic carbon, thereby contributing to improved soil fertility. SOM, as the primary reservoir of organic carbon in soil, serves as the fundamental substrate for these enzymatic activities. In this study, the activities of urease, sucrase, CAT, and acid phosphatase were observed to decrease progressively with increasing Sb concentration in the soil. In contrast, inoculation with *Cupriavidus* sp. S-8-2 significantly enhanced enzyme activities compared to non-inoculation treatments (Table 2), which is consistent with previous findings demonstrating the capacity of PGPR to stimulate soil enzyme activities (Li X. et al., 2020). This enhancement may be attributable to PGPR-mediated modifications in rhizosphere microbial activity, improvements in soil physicochemical properties (e.g., increased TN content), and the mitigation of soil toxicity (Ju et al., 2019; Zhou et al., 2022). Soil urease, a key enzyme in the nitrogen cycle, showed significant positive correlations with TN and SOM content (Figure 5), supporting its role in the conversion of organic nitrogen into plant-available ammonium-N (Cheng et al., 2025). This aligns with documented positive association between urease activity and soil nutrient content levels (Fu et al., 2025). Similarly, acid phosphatase facilitates phosphorus mineralization, thereby directly influencing soil-available phosphorus and alleviating phosphorus limitation under stressful environments conditions (Zhu et al., 2018), as evidenced by the present results. PGPR have been found to enhance CAT activity, as demonstrated in *Gossypium hirsutum* L. (Qureshi et al., 2019), as well as sucrase and urease activities in HM-contaminated soils through strains like *Bacillus* sp. ZC3-2-1 (Liu et al., 2022). CAT enzyme contributes to HM detoxification by catalyzing the decomposition of toxic H₂O₂ into less harmful products and is also involved in soil carbon cycling (Das and Sen, 2024). Therefore, inoculation with *Cupriavidus* sp. S-8-2 enhances nitrogen and phosphorus cycling through the activation of specific enzymes, leading to improved soil properties and enhanced plant growth under Sb stress. Furthermore, the pronounced negative correlation observed between urease activity and Sb concentration underscores the urease activity could serve as a potential indicator for evaluating Sb toxicity in soil ecosystems. Further investigation into this correlation may elucidate the mechanisms underlying Sb-induced enzymatic inhibition and its broader ecological implications.

### 4.3 Inoculation with *Cupriavidus* sp. S-8-2 modulates the structure, functionality, and co-occurrence network of rhizosphere microbiota in pepper plants under Sb stress

Rhizosphere biochemical properties can influence microbial community structure, which in turn regulate plant growth and HM uptake through diverse mechanisms (Solomon et al., 2024). PGPR treatments have been shown to critically shape bacterial diversity under HM exposure conditions (Hu et al., 2021; Muratova et al., 2023). A significant decrease in alpha diversity (Shannon index) was observed following inoculation under 500 mg/kg Sb stress (*p* < 0.05, Supplementary Figure S2B). These results suggest that inoculation with *Cupriavidus* sp. S-8-2 promoted a rapid response of rhizosphere microorganisms to Sb stress by competitive exclusion of taxa with lower Sb tolerance, while concurrently enriching HM-tolerant families such as *Rhizobiaceae* and *Sphingomonadaceae*. This restructuring of microbial community may contribute to enhanced plant survival under adverse conditions. Supporting this hypothesis, PCoA analysis (Supplementary Figure S3) reveals potential structural changes in the rhizosphere microbial community that could be associated with altered rhizosphere toxicity dynamics. Crucially, inoculation significantly modulated the relative abundance of several bacterial taxa involved in HM resistance and plant growth promotion. These included the phyla Proteobacteria and Firmicutes, as well as genera such as *Ramlibacter*, *Brevundimonas*, *Pseudomonas*, *Microvirga*, *Dyella*, and *Nitrospira*. An increase in the relative abundance of Proteobacteria was observed under elevated Sb stress when inoculation, which is consistent with earlier reports in Cd-stressed wheat (Zhao et al., 2024). This phylum is known for its intrinsic HM tolerance mediated by detoxification enzymes and regulatory proteins that enhance its competitive advantage in contaminated environments (Bai et al., 2022). Moreover, Proteobacteria include genera key genera, such as *Rhodanobacter* and *Luteimonas,* which have been linked to oxidative stress mitigation through enzymatic ROS detoxification (Caldeira et al., 2021). These observations are consistent with our findings showing reduced MDA levels in inoculated roots (Figure 2). Despite their recognized role in SOM decomposition and nutrient cycling, the abundance of Proteobacteria showed a significant negative correlation with SOM content (Figure 5B). Many members of Proteobacteria also exhibit PGP traits, including symbiotic nitrogen fixation (Li P. et al., 2024; Shi et al., 2023), which may improve nutrient availability for plants. Other enriched genera, such as *Ramlibacter*, *Pseudomonas*, and *Dyella* have previously been implicated in HM resistance and PGP capabilities (Zhao et al., 2024; Shi et al., 2023; Cai et al., 2023). Similarly, *Microvirga* has been associated with HM resistance and nitrogen cycling processes (Zhang N. et al., 2025). The inoculation-induced enhancement of nutrient availability under Sb stress may have facilitated the proliferation of copiotrophic microbial groups, such as Proteobacteria and Firmicutes, potentially leading to a decrease in the relative abundance of oligotrophic taxa, including Chloroflexi and Acidobacteria (Figure 3A). This trend was further corroborated by LefSe analysis, which identified Proteobacteria as significant discriminative biomarkers in inoculated treatments subjected to high Sb stress, while Chloroflexi were more prominently associated with non-contaminated controls (Figure 3C). Within the rhizosphere of pepper inoculated with *Cupriavidus* sp. S-8-2, *Rhizobiaceae* and *Sphingomonadaceae* were identified as key biomarkers under 500 mg/kg and 1,000 mg/kg Sb stress, respectively. *Rhizobiaceae* harbors essential genes involved in Sb(III) oxidation, N₂ fixation, and carbon fixation (Li Y. et al., 2022), whereas *Sphingomonadaceae* has been reported to exhibit increased abundance in response to Cd exposure in previous studies (Zhou et al., 2019). Collectively, these findings suggest that inoculation with *Cupriavidus* sp. S-8-2 may help shape the rhizosphere microbiome under Sb contamination by enriching bacterial taxa associated with HM tolerance and nutrient cycling, potentially contributing to enhanced plant growth.

The network analysis of microbial co-occurrence provides a powerful framework for elucidating complex microbial interactions beyond conventional sample-level comparisons, particularly in extreme environments (Guo et al., 2022; Ma et al., 2020; Mercado et al., 2022). In this study, inoculation with *Cupriavidus* sp. S-8-2 was found to significantly enhance node connectivity and mean degree in rhizosphere bacterial networks, especially under severe Sb stress (1,000 mg/kg). This increase in structural complexity, as reported by Wang J. et al. (2023), may indicate an improved capacity for adaptation to environmental stress (Xing et al., 2024). Interestingly, the microbial network, whether under individual Sb Stress or PGPR inoculation, showed an increased proportion of negative correlations (Table 2). Such shifts in correlation patterns may reflect dynamic adjustments in both symbiotic and competitive relationships among microbial taxa, as previously observed under nutrient-limited stress conditions (Anas et al., 2025). These competitive interactions warrant further investigation under Sb-induced stress conditions.

Key taxa within microbial networks under PGPR treatment were identified as functional groups involved in nutrient cycling (Liu et al., 2024; Chi et al., 2025), potentially contributing to the structuring microbial co-occurrence patterns (Ding et al., 2023). Several keystone taxa, including Proteobacteria, *Rhodanobacter*, *Ramlibacter,* and *Luteibacter*, were identified, all of which have been previously associated with HM removal and detoxification (Peng et al., 2022). Notably, *Ramlibacter* has been shown to enhance phosphorus solubilization and promote ryegrass growth (Zhao et al., 2024). The emergence of *Rhodanobacter* as a keystone hub under high Sb stress (Figure 4) is consistent with its previously reported capacity for Sb(III) oxidation in rhizosphere systems (Kataoka et al., 2018). This oxidation mechanism contributes to the alleviation of Sb-induced ROS generation, a process that has been mechanistically elucidated by Zhang Y. et al. (2025). Inoculation with *Cupriavidus* sp. S-8-2 was observed to modify the rhizosphere co-occurrence network structure (Table 2), thereby enhancing functional stability, as indicated by increased modularity and reduced the number of positive correlations (Coyte et al., 2015).

Although inoculation with *Cupriavidus* sp. S-8-2 did not significantly alter the overall structure of the rhizosphere microbiota, functional predictions generated using PICRUSt2 revealed notable modifications in specific secondary metabolic pathways (Supplementary Figure S4). In particular, TCA cycle, a central metabolic pathway that supplies energy and anabolic precursors essential for cellular proliferation and survival (MacLean et al., 2023), exhibited mitigated inhibition following inoculation. Given the reported synergistic relationship between glycolysis and TCA cycle under Al exposure (Guan et al., 2024), these findings suggest that *Cupriavidus* sp. S-8-2 may help alleviate energy supply deficits under Sb stress (Supplementary Figure S4). Inoculated treatments under high Sb stress (1,000 mg/kg) also showed increased activity in pathways associated with aromatic amino acid biosynthesis. Aromatic amino acids play key roles in regulating auxin signaling and serve as precursors for the biosynthesis of antioxidant phenolic compounds (Samsami and Maali-Amiri, 2024) which is consistent with the observed increase in total flavonoid content. Specifically, tyrosine-derived flavonoids have been shown to enhance plant tolerance to abiotic stress through stress-induced accumulation (Sanches Silva et al., 2020). Additionally, inoculated systems exhibited significantly improved aldehyde degradation, linked to IAA production, and enhanced carboxylate metabolism, associated with ACC deaminase synthesis. Aldehyde degradation is biochemically driven by aldehyde dehydrogenase, which converts indole-3-acetaldehyde into IAA—the final step in auxin biosynthesis (Zhang et al., 2023). Carboxylate metabolism produces *α*-ketobutyrate, a key co-substrate for ACC deaminase synthesis, which helps regulate ethylene-mediated stress responses (Gupta et al., 2022). Both processes have been shown to improve root growth and stress tolerance, as confirmed by our earlier research (Zheng et al., 2023). Pathways related to glutathione metabolism, known for their roles in pollutant sequestration and antioxidant defense mechanisms (Dorion et al., 2021), were also significantly upregulated under Sb stress following inoculation with *Cupriavidus* sp. S-8-2. The metabolic changes observed after inoculation with *Cupriavidus* sp. S-8-2 were correlated with reduced Sb accumulation in plants and enhanced growth under stress conditions. This functional reconfiguration of metabolic processes likely enhanced Sb tolerance and supported host plant acclimation. Future studies should monitor the persistence of *Cupriavidus* sp. S-8-2 in soil environments to validate its ecological role.

### 4.4 Mechanisms underlying the regulation of Sb uptake and accumulation in pepper plants by *Cupriavidus* sp. S-8-2 inoculation under Sb stress

PGPR-mediated modulation of HM uptake involves a series of interconnected processes encompassing plant physiology, soil HM bioavailability, and intraplant transport mechanisms (Sultana et al., 2024; Qin et al., 2024). Integrative analyses (RDA, Pearson correlation, Mantel tests, and SEM; Figures 5, 6) revealed that soil physicochemical properties directly influenced pepper biomass and the distribution of Sb in plant tissues, thereby governing Sb accumulation patterns under Sb stress conditions.

In this study, *Cupriavidus* sp. S-8-2 inoculation directly enhanced plant growth by improving the availability of soil nutrients (TN, TP, SOM). The resulting changes in N and P availability, along with shifts in soil pH, rapidly modulated soil enzyme activities, thereby altering the structure of the soil microbial community. The inoculation treatments enriched HM-tolerant genera, such as *Gaiella*, *Nitrospira*, *JG30-KF-AS9*, *Haliangium*, which are adapted to oligotrophic conditions and exhibited positive correlations with plant biomass under Sb stress. Genus *Gaiella* has been previously reported to exhibit notable HM tolerance (Wu et al., 2024) and to promote plant growth in contaminated soils (Li Y. et al., 2022; Li Z. et al., 2022; Wu et al., 2024). While *JG30-KF-AS9* is associated with the mineralization of soil organic carbon (Cao et al., 2025). *Nocardioides* is known to produce phytohormones and perform nitrogen fixation under HM stress (Meena et al., 2020), whereas members of the *Allorhizobium-Neorhizobium-Pararhizobium-Rhizobium* complex are capable of degrading xenobiotics and simultaneously fixing nitrogen (Talwar and Chatli, 2020). Additionally, rare genus like *Pseudarthrobacter* encodes genes involved in auxin biosynthesis and atmospheric N₂ fixation (Li X. et al., 2020), further supporting the role of inoculation in recruiting functionally diverse bacterial taxa to improve plant fitness under Sb stress. Significant positive correlations were observed between microbial community composition and soil enzyme activities. Notably, the abundance of *Proteobacteria* was positively correlated with sucrase activity and SOM content, which aligns with the metabolic versatility of this phylum in organically enriched soils.

PGPR application has been shown to reduce the entry of HMs into food chains, with plant uptake primarily governed by bioavailability, which is modulated by soil pH and SOM content (Li J. et al., 2024; Li P. et al., 2024). Although tissue Sb concentrations increased with elevated Sb exposure levels, inoculation with PGPR significantly reduced root Sb accumulation (*p* < 0.01), leading to reduced concentrations in leaves and fruits. This reduction may help mitigate the risk of secondary pollution and improve food safety in Sb-contaminated environments. These findings are consistent with previous observations of PGPR-induced decreases in HM bioavailability in ryegrass (Ke et al., 2021). However, some PGPR consortia, such as *Pseudomonas aeruginosa* have been reported to enhance HM uptake (Shi et al., 2022), highlighting the functional variability among these PGPR strains. The observed reduction in Sb content in pepper plants may be due to the superior phosphate solubilizing ability and the secretion of low-molecular-weight acids by *Cupriavidus* sp. S-8-2 under Sb stress, which could potentially immobilize Sb through precipitation reactions (Zheng et al., 2023). Additionally, rhizosphere SOM has been shown to facilitate HM binding, thereby reducing Sb bioavailability (Wang et al., 2024), with the efficiency of PGPR being closely related to SOM content (Bai et al., 2024). Moreover, elevated TP levels can compete for soil binding sites, thus decreasing Sb mobilization (Yan et al., 2017; Lisac et al., 2019). These mechanisms were concurrently observed under high Sb stress (1,000 mg/kg), as inoculation not only increased TP and SOM levels but also reduced root Sb accumulation. Moreover, enhanced root growth (Table 1) contributed to enhanced tolerance to Sb and decreased its translocation to above-ground tissues.

Furthermore, inoculation with *Cupriavidus* sp. S-8-2 was found to enhance the abundance of certain microbial taxa that exhibited negative correlations with Sb concentrations (Figure 5). For instance, the genus *Gemmatimonas* showed an inverse relationship with Sb bioavailability, suggesting its involvement in Sb immobilization (Li et al., 2021). Conversely, *Flavobacterium* showed a strong positive correlation with Sb accumulation in plant roots, which may be attributed to its HM resistance mechanisms involving oxidation and methylation processes (Sun et al., 2023). Additionally, *Dyella* has been reported to participate in the cycling of Al and iron ions under oligotrophic conditions (Chen et al., 2023). Notably, inoculation led to a decrease in the relative abundance of Patescibacteria, a phylum associated with HM activation and migration (Tian et al., 2022). These observations are consistent with previous studies indicating that Proteobacteria, Actinobacteria, Bacteroidetes, Firmicutes, and Gemmatimonadetes are frequently associated with variations in HM accumulation patterns in plants (Wang X. et al., 2023). Halim et al. (2025) demonstrated that PGPR employ multiple mechanisms, including biosorption, bioaccumulation, plant growth promotion, organic acids secretion, siderophore enhancement, and extracellular polymer synthesis to immobilize Cd at the soil–plant interface, thereby restricting Cd translocation to above-ground plant tissues. Collectively, *Cupriavidus* sp. S-8-2 contributes to the enrichment of functionally diverse microbial communities in the rhizosphere of pepper plant, including phosphorus-solubilizing bacteria, HM-immobilizing microorganisms, plant growth-promoting rhizobacteria, and nitrogen-cycling bacteria. These functionally distinct microbial groups may work in concert to decrease Sb bioavailability through immobilization processes, while simultaneously supporting plant growth under Sb stress conditions.

Beyond providing preliminary mechanistic insights, this study highlights the practical applications of sustainable remediation strategies for Sb-contaminated environments. *Cupriavidus* sp. S-8-2 demonstrates potential as a bioaugmentation agent or biofertilizer in agricultural areas affected by Sb contamination, supported by a 54.75% reduction in Sb accumulation in fruits and 18.48% increase in fruits biomass, even under a high Sb stress level of 1,000 mg/kg in pot experiments. However, further targeted investigations are required to validate the underlying mechanisms. The transition from laboratory to field application necessitates confirmation of the strain’s persistence in non-sterile soil environments and field validation of its bioaugmentation efficacy, both of which are essential for the development of precision bioformulations tailored for Sb-affected agricultural systems.

## 5 Conclusion

This study demonstrates that inoculation with *Cupriavidus* sp. S-8-2 significantly enhances growth performance and Sb stress tolerance in *Capsicum annuum* L. cultivated in Sb-contaminated soils. Application of this PGPR resulted in increased plant biomass, enhanced key soil enzymatic functions, and notable restructuring of the rhizosphere bacterial communities. Furthermore, PGPR inoculation improved nutrient availability in the rhizosphere while mitigating Sb phytotoxicity in aerial tissues, primarily through reduced Sb accumulation in roots and restricted translocation to shoots. Importantly, *Cupriavidus* sp. S-8-2 facilitated the enrichment of beneficial microbial taxa, particularly members of the phylum Proteobacteria, which are known for their ecological adaptability and functional dominance, potentially contributing to enhanced plant resistance to Sb stress. Integrated analysis of these findings provides mechanistic insights into the role of rhizosphere PGPR application in alleviating HM stress and highlights their potential application as biofertilizers to reduce HM accumulation in agricultural systems. Future research should focus on elucidating the molecular mechanisms governing rhizobacterial responses to HM stress and characterizing root exudate profiles through integrated metagenomic and metabolomic approaches. In parallel, field-based validation studies are necessary to evaluate the capability of *Cupriavidus* sp. S-8-2 to promote plant growth and enhance Sb phytoremediation across different plant species and multi-metal contaminated field soils. Such efforts will contribute to the effective and sustainable application of PGPR as a bioaugmentation agent or biofertilizer in agricultural areas affected by Sb contamination, contributing to the advancement of sustainable agricultural practices.

## Data Availability

The sequences data reported in this study have been deposited in NCBI SRA with the accession number PRJNA1313877.

## References

[ref9001] Abdelkrim S. Jebara S. H. Saadani O. Abid G. Taamalli W. Zemni H.. (2020). In situ effects of Lathyrus sativus-PGPR to remediate and restore quality and fertility of Pb and Cd polluted soils[J]. Ecotoxicol. Environ. Saf. 192:110260. doi: 10.1016/j.ecoenv.2020.110260 32050135

[ref2] Anas M. Khalid A. Saleem M. H. Ali Khan K. Ahmed Khattak W. Fahad S. (2025). Symbiotic synergy: unveiling plant-microbe interactions in stress adaptation. J. Crop Health 77, 1–21. doi: 10.1007/s10343-024-01070-z, PMID: 40881164

[ref9002] Andriyanto S. Aryati Y. Sumiati T. Lusiastuti A. M. Nurhidayat Kurniawan K.. (2022). The potential roles of gut microbiome in modulating the immune response of Asian Redtail catfish (Hemibagrus nemurus) vaccinated with Aeromonas hydrophila[J]. HAYATI J. Biosci. 29, 266–278. doi: 10.4308/hjb.29.3.266-278

[ref9003] Aziz M. A. Adil B. Ali I. Alghamdi A. G. (2025). Role of biochar and PGPR in improving soil biochemical characteristics and maize growth under Cr contamination[J]. Int. J. Phytoremediation. 27, 1154–1168. doi: 10.1080/15226514.2025.2485302 40170427

[ref4] Bai X. Bol R. Chen H. Cui Q. Qiu T. Zhao S.. (2024). A meta-analysis on crop growth and heavy metals accumulation with PGPB inoculation in contaminated soils. J. Hazard. Mater. 471:134370. doi: 10.1016/j.jhazmat.2024.134370, PMID: 38688214

[ref5] Bai Y. Su J. Ali A. Chang Q. Gao Z. Wang Y.. (2022). Insights into the mechanism of Mn (II)-based autotrophic denitrification: performance, genomic, and metabonomics. Sci. Total Environ. 810:151185. doi: 10.1016/j.scitotenv.2021.151185, PMID: 34699810

[ref6] Bhardwaj T. Kour J. Chouhan R. Devi K. Singh H. Gandhi S. G.. (2024). Integrated transcriptomic and physio-molecular studies unveil the melatonin and PGPR induced protection to photosynthetic attributes in *Brassica juncea* L. under cadmium toxicity. J. Hazard. Mater. 476:134875. doi: 10.1016/j.jhazmat.2024.134875, PMID: 38936187

[ref7] Cai P. Chen Q. Du W. Yang S. Li J. Cai H.. (2023). Deciphering the dynamics of metal and antibiotic resistome profiles under different metal (loid) contamination levels. J. Hazard. Mater. 455:131567. doi: 10.1016/j.jhazmat.2023.131567, PMID: 37167868

[ref8] Caldeira J. B. Chung A. P. Morais P. V. Branco R. (2021). Relevance of FeoAB system in Rhodanobacter sp. B2A1Ga4 resistance to heavy metals, aluminium, gallium, and indium. Appl. Microbiol. Biotechnol. 105, 3301–3314. doi: 10.1007/s00253-021-11254-6, PMID: 33791837

[ref9] Cao R. Yang J. Meng Z. Zhou H. Dong X. Peng L.. (2025). Linking microbial communities and organic matter dynamics in Longjing and Fuding tea ecosystems. J. Food Biochem. 2025:9981444. doi: 10.1155/jfbc/9981444

[ref10] Chaudhary P. Xu M. Ahamad L. Chaudhary A. Kumar G. Adeleke B. S.. (2023). Application of synthetic consortia for improvement of soil fertility, pollution remediation, and agricultural productivity: a review. Agronomy 13:643. doi: 10.3390/agronomy13030643

[ref11] Chen Y. Fu W. Xiao H. Zhai Y. Luo Y. Wang Y.. (2023). A review on rhizosphere microbiota of tea plant (*Camellia sinensis* L): recent insights and future perspectives. J. Agric. Food Chem. 71, 19165–19188. doi: 10.1021/acs.jafc.3c02423, PMID: 38019642

[ref12] Cheng Z. Liu W. Li Z. Carpio M. J. García-Gil J. C. Wang Z.. (2025). Plant litter crust enhances nitrogen accumulation by regulating microbial diversity and urease activity in semi-arid sandy soils. Appl. Soil Ecol. 205:105774. doi: 10.1016/j.apsoil.2024.105774

[ref13] Chi Y. Ma X. Chu S. You Y. Chen X. Wang J.. (2025). Nitrogen cycle induced by plant growth-promoting rhizobacteria drives “microbial partners” to enhance cadmium phytoremediation. Microbiome 13, 113–119. doi: 10.1186/s40168-025-02113-x, PMID: 40329393 PMC12054286

[ref14] Coyte K. Z. Schluter J. Foster K. R. (2015). The ecology of the microbiome: networks, competition, and stability. Science 350, 663–666. doi: 10.1126/science.aad2602, PMID: 26542567

[ref15] Das P. Sen P. Relevance of oxidoreductases in cellular metabolism and defence. Reactive Oxygen Species-Advances and Developments. Intech Open, (2024). Available online at: 10.5772/intechopen.112302 (Accessed May 15, 2025).

[ref16] Daunoras J. Kačergius A. Gudiukaitė R. (2024). Role of soil microbiota enzymes in soil health and activity changes depending on climate change and the type of soil ecosystem. Biology 13:85. doi: 10.3390/biology13020085, PMID: 38392304 PMC10886310

[ref17] Ding N. Ullah H. Yu G. He Y. Liu L. Xie Y.. (2023). Spatial dynamics of pH in the rhizosphere of *Leersia hexandra* Swartz at different chromium exposure. Ecotoxicol. Environ. Saf. 263:115380. doi: 10.1016/j.ecoenv.2023.115380, PMID: 37597293

[ref18] Dorion S. Ouellet J. C. Rivoal J. (2021). Glutathione metabolism in plants under stress: beyond reactive oxygen species detoxification. Meta 11:641. doi: 10.3390/metabo11090641, PMID: 34564457 PMC8464934

[ref19] Dutta B. Deka K. Das B. K. Borah J. Saloi H. J. Medhi B. K.. (2025). Soil pollution and remediation strategies: new approaches for soil quality improvement. J. Adv. Biol. Biotechnol. 28, 962–992. doi: 10.9734/jabb/2025/v28i52358

[ref20] El-Ballat E. M. Elsilk S. E. Ali H. M. Ali H. E. Hano C. El-Esawi M. A. (2023). Metal-resistant PGPR strain *Azospirillum brasilense* EMCC1454 enhances growth and chromium stress tolerance of chickpea (*Cicer arietinum* L.) by modulating redox potential, osmolytes, antioxidants, and stress-related gene expression. Plants 12:2110. doi: 10.3390/plants12112110, PMID: 37299089 PMC10255068

[ref21] El-Meihy R. M. Abou-Aly H. E. Youssef A. M. Tewfike T. A. El-Alkshar E. A. (2019). Efficiency of heavy metals-tolerant plant growth promoting bacteria for alleviating heavy metals toxicity on sorghum. Environ. Exp. Bot. 162, 295–301. doi: 10.1016/j.envexpbot.2019.03.005

[ref22] Espinola E. C. Cabreros M. M. N. Redillas M. C. F. R. (2025). Morpho-physiological adaptations of rice cultivars under heavy metal stress: a systematic review and meta-analysis. Life 15:189. doi: 10.3390/life15020189, PMID: 40003598 PMC11856324

[ref23] Feng R. Wei C. Tu S. Ding Y. Wang R. Guo J. (2013). The uptake and detoxification of antimony by plants: a review. Environ. Exp. Bot. 96, 28–34. doi: 10.1016/j.envexpbot.2013.08.006

[ref24] Fu Y. Yu Y. Yang S. Yang G. Huang H. Yang Y.. (2025). Effects of fertilization on soil physicochemical properties and enzyme activities of *Zanthoxylum planispinum* var. dingtanensis plantation. Forests 16:418. doi: 10.3390/f16030418

[ref26] Guan J. Zhang Y. Li D. Shan Q. Hu Z. Chai T.. (2024). Synergistic role of phenylpropanoid biosynthesis and citrate cycle pathways in heavy metal detoxification through secretion of organic acids. J. Hazard. Mater. 476:135106. doi: 10.1016/j.jhazmat.2024.135106, PMID: 38970974

[ref9004] Guo B. Zhang L. Sun H. Gao M. Yu N. Zhang Q.. (2022). Microbial co-occurrence network topological properties link with reactor parameters and reveal importance of low-abundance genera[J]. NPJ Biofilms Microbiomes. 8:3. doi: 10.1038/s41522-021-00263-y 35039527 PMC8764041

[ref27] Gupta R. Khan F. Alqahtani F. M. Hashem M. Ahmad F. (2024). Plant growth–promoting Rhizobacteria (PGPR) assisted bioremediation of heavy metal toxicity. Appl. Biochem. Biotechnol. 196, 2928–2956. doi: 10.1007/s12010-023-04545-3, PMID: 37097400

[ref28] Gupta A. Singh K. Charles M. Pathak N. (2022). Role of ACC deaminase producing plant growth promoting rhizobacteria in ameliorating the salinity stress conditions: a review. Era's J. Med. Res. 9, 60–77. doi: 10.24041/EJMR2022.09

[ref29] Haider F. U. Zulfiqar U. ul Ain N. Mehmood T. Ali U. Aguila L. C. R.. (2024). Managing antimony pollution: insights into soil–plant system dynamics and remediation strategies. Chemosphere 362:142694. doi: 10.1016/j.chemosphere.2024.142694, PMID: 38925521

[ref30] Halim M. A. Rahman M. M. Mallavarapu M. Naidu R. (2025). Rhizo-immobilization of cd by plant growth promoting Rhizobacteria-A review. Food Toxic. Saf., 207–223. doi: 10.1007/978-981-96-4128-4_9

[ref31] Hu J. Yang T. Friman V. P. Kowalchuk G. A. Hautier Y. Li M.. (2021). Introduction of probiotic bacterial consortia promotes plant growth via impacts on the resident rhizosphere microbiome. Proc. R. Soc. Lond. B Biol. Sci. 288:20211396. doi: 10.1098/rspb.2021.1396, PMID: 34641724 PMC8511750

[ref32] Huang Q. Meng F. Chen W. Cai Y. Xiao E. (2025). Biochar influences the transformation and translocation of antimony in the rhizosphere–rice system. Toxics 13:389. doi: 10.3390/toxics13050389, PMID: 40423469 PMC12115445

[ref33] Hussain A. Shah M. Hamayun M. Iqbal A. Qadir M. Alataway A.. (2023). Phytohormones producing rhizobacteria alleviate heavy metals stress in soybean through multilayered response. Microbiol. Res. 266:127237. doi: 10.1016/j.micres.2022.127237 36270107

[ref34] Ju W. Liu L. Fang L. Cui Y. Duan C. Wu H. (2019). Impact of co-inoculation with plant-growth-promoting rhizobacteria and rhizobium on the biochemical responses of alfalfa-soil system in copper contaminated soil. Ecotoxicol. Environ. Saf. 167, 218–226. doi: 10.1016/j.ecoenv.2018.10.016, PMID: 30342354

[ref35] Kangi E. (2024). Investigating Changing Macronutrient Dynamics at a Plant, Microbe and Plant-Microbe Interactions Scale. West Virginia University. Available online at: https://researchrepository.wvu.edu/etd/12526 (accessed May 18, 2025).

[ref36] Karimi N. Pakdel H. Souri Z. Norouzi L. Rizwan M. Yong J. W. H. (2025). Effects of phytostabilized zinc sulfide nanocomposites on growth and arsenic accumulation in wheat (*Triticum aestivum* L.) under arsenic stress. Plant Stress 16:100886. doi: 10.1016/j.stress.2025.100886

[ref37] Kataoka T. Mitsunobu S. Hamamura N. (2018). Influence of the chemical form of antimony on soil microbial community structure and arsenite oxidation activity. Microbes Environ. 33, 214–221. doi: 10.1264/jsme2.ME17182, PMID: 29887548 PMC6031390

[ref38] Ke T. Guo G. Liu J. Zhang C. Tao Y. Wang P.. (2021). Improvement of the cu and cd phytostabilization efficiency of perennial ryegrass through the inoculation of three metal-resistant PGPR strains. Environ. Pollut. 271:116314. doi: 10.1016/j.envpol.2020.116314, PMID: 33360656

[ref39] Khanna K. Jamwal V. L. Kohli S. K. Gandhi S. G. Ohri P. Bhardwaj R.. (2019a). Role of plant growth promoting bacteria (PGPRs) as biocontrol agents of *Meloidogyne incognita* through improved plant defense of *Lycopersicon esculentum*. Plant Soil 436, 325–345. doi: 10.1007/s11104-019-03932-2

[ref40] Khanna K. Kohli S. K. Ohri P. Bhardwaj R. al-Huqail A. A. Siddiqui M. H.. (2019b). Microbial fortification improved photosynthetic efficiency and secondary metabolism in *Lycopersicon esculentum* plants under Cd stress. Biomolecules 9:581. doi: 10.3390/biom9100581, PMID: 31591372 PMC6843591

[ref41] Klindworth A. Pruesse E. Schweer T. Peplies J. Quast C. Horn M.. (2013). Evaluation of general 16S ribosomal RNA gene PCR primers for classical and next-generation sequencing-based diversity studies. Nucleic Acids Res. 41:e1. doi: 10.1093/nar/gks808, PMID: 22933715 PMC3592464

[ref42] Kong L. Hu X. Peng X. He M. (2024). Securing the global antimony supply chain. Science 386:281. doi: 10.1126/science.adt1636, PMID: 39418364

[ref43] Kong Z. Wu Z. Glick B. R. He S. Huang C. Wu L. (2019). Co-occurrence patterns of microbial communities affected by inoculants of plant growth-promoting bacteria during phytoremediation of heavy metal-contaminated soils. Ecotoxicol. Environ. Saf. 183:109504. doi: 10.1016/j.ecoenv.2019.109504, PMID: 31421537

[ref44] Laha A. Sengupta S. Bhattacharyya S. Bhattacharyya K. GuhaRoy S. (2024). Isolation and characterization of rhizobacteria from lentil for arsenic resistance and plant growth promotion. 3 Biotech 14:30. doi: 10.1007/s13205-023-03873-9, PMID: 38178896 PMC10761649

[ref45] Li X. Fu W. Meng F. Guan C. (2022). Carotenoid-mediated regulation of photosynthetic performance and antioxidant defense confer tolerance to perfluorooctanoic acid stress in *Nicotiana tabacum*. Plant Growth Regul. 98, 307–319. doi: 10.1007/s10725-022-00856-3

[ref46] Li Y. Guo L. Kolton M. Yang R. Zhang M. Qi F.. (2022). Chemolithotrophic biological nitrogen fixation fueled by antimonite oxidation may be widespread in Sb-contaminated habitats. Environ. Sci. Technol. 57, 231–243. doi: 10.1021/acs.est.2c06424, PMID: 36525577

[ref47] Li H. Qiu Y. Yao T. Ma Y. Zhang H. Yang X. (2020). Effects of PGPR microbial inoculants on the growth and soil properties of *Avena sativa*, *Medicago sativa*, and *Cucumis sativus* seedlings. Soil Tillage Res. 199:104577. doi: 10.1016/j.still.2020.104577

[ref48] Li P. Tian Y. Yang K. Tian M. Zhu Y. Chen X.. (2024). Mechanism of microbial action of the inoculated nitrogen-fixing bacterium for growth promotion and yield enhancement in rice (*Oryza sativa* L.). Adv. Biotechnol. 2:32. doi: 10.1007/s44307-024-00038-4, PMID: 39883349 PMC11709144

[ref49] Li B. Xu R. Sun X. Han F. Xiao E. Chen L.. (2021). Microbiome–environment interactions in antimony-contaminated rice paddies and the correlation of core microbiome with arsenic and antimony contamination. Chemosphere 263:128227. doi: 10.1016/j.chemosphere.2020.128227, PMID: 33297183

[ref50] Li J. Xu Y. Zhang Y. Liu Z. Gong H. Fang W.. (2024). Quantifying the mitigating effect of organic matter on heavy metal availability in soils with different manure applications: a geochemical modelling study. Ecotoxicol. Environ. Saf. 276:116321. doi: 10.1016/j.ecoenv.2024.116321, PMID: 38608382

[ref51] Li X. Yao S. Bian Y. Jiang X. Song Y. (2020). The combination of biochar and plant roots improves soil bacterial adaptation to PAH stress: insights from soil enzymes, microbiome, and metabolome. J. Hazard. Mater. 400:123227. doi: 10.1016/j.jhazmat.2020.123227, PMID: 32585520

[ref52] Li Z. Zheng Y. Li Y. Cheng X. Huang S. Yang X.. (2022). Genotype-specific recruitment of rhizosphere bacteria from sandy loam soil for growth promotion of *Cucumis sativus* var. *hardwickii*. Front. Microbiol. 13:910644. doi: 10.3389/fmicb.2022.910644, PMID: 35832804 PMC9271904

[ref53] Lisac K. Topić F. Arhangelskis M. Cepić S. Julien P. A. Nickels C. W.. (2019). Halogen-bonded cocrystallization with phosphorus, arsenic and antimony acceptors. Nat. Commun. 10:61. doi: 10.1038/s41467-018-07957-6, PMID: 30610194 PMC6320372

[ref54] Liu L. Gao Y. Yang W. Liu J. Wang Z. (2024). Community metagenomics reveals the processes of nutrient cycling regulated by microbial functions in soils with P fertilizer input. Plant Soil 499, 139–154. doi: 10.1007/s11104-023-05875-1

[ref56] Liu A. Wang W. Zheng X. Chen X. Fu W. Wang G.. (2022). Improvement of the cd and Zn phytoremediation efficiency of rice (*Oryza sativa*) through the inoculation of a metal-resistant PGPR strain. Chemosphere 302:134900. doi: 10.1016/j.chemosphere.2022.134900, PMID: 35568210

[ref58] Luo H. Win C. S. Lee D. H. He L. Yu J. M. (2024). *Microbacterium azadirachtae* CNUC13 enhances salt tolerance in maize by modulating osmotic and oxidative stress. Biology 13:244. doi: 10.3390/biology13040244, PMID: 38666856 PMC11048422

[ref59] Ma B. Wang Y. Ye S. Liu S. Stirling E. Gilbert J. A.. (2020). Earth microbial co-occurrence network reveals interconnection pattern across microbiomes. Microbiome 8, 82–12. doi: 10.1186/s40168-020-00857-2, PMID: 32498714 PMC7273686

[ref60] MacLean A. Legendre F. Appanna V. D. (2023). The tricarboxylic acid (TCA) cycle: a malleable metabolic network to counter cellular stress. Crit. Rev. Biochem. Mol. Biol. 58, 81–97. doi: 10.1080/10409238.2023.2201945, PMID: 37125817

[ref61] Mansoor S. Ali A. Kour N. Bornhorst J. AlHarbi K. Rinklebe J.. (2023). Heavy metal induced oxidative stress mitigation and ROS scavenging in plants. Plants 12:3003. doi: 10.3390/plants12163003, PMID: 37631213 PMC10459657

[ref62] Meena K. K. Bitla U. M. Sorty A. M. Singh D. P. Gupta V. K. Wakchaure G. C.. (2020). Mitigation of salinity stress in wheat seedlings due to the application of phytohormone-rich culture filtrate extract of methylotrophic actinobacterium *Nocardioides* sp. NIMMe6. Front. Microbiol. 11:2091. doi: 10.3389/fmicb.2020.02091 33071995 PMC7531191

[ref63] Mercado J. V. Koyama M. Nakasaki K. (2022). Co-occurrence network analysis reveals loss of microbial interactions in anaerobic digester subjected to repeated organic load shocks. Water Res. 221:118754. doi: 10.1016/j.watres.2022.118754, PMID: 35759844

[ref64] Moustaka J. Sperdouli I. Panteris E. Adamakis I. D. S. Moustakas M. (2025). Aspirin foliar spray-induced changes in light energy use efficiency, chloroplast ultrastructure, and ROS generation in tomato. Int. J. Mol. Sci. 26:1368. doi: 10.3390/ijms26031368, PMID: 39941138 PMC11818874

[ref65] Muratova A. Golubev S. Romanova V. Sungurtseva I. Nurzhanova A. (2023). Effect of heavy-metal-resistant PGPR inoculants on growth, rhizosphere microbiome and remediation potential of *miscanthus*× *giganteus* in zinc-contaminated soil. Microorganisms 11:1516. doi: 10.3390/microorganisms11061516, PMID: 37375018 PMC10304086

[ref66] Ou K. He X. Cai K. Zhao W. Jiang X. Ai W.. (2022). Phosphate-solubilizing *pseudomonas* sp. strain WS32 rhizosphere colonization-induced expression changes in wheat roots. Front. Microbiol. 13:927889. doi: 10.3389/fmicb.2022.927889, PMID: 35847091 PMC9279123

[ref67] Park Y. G. Mun B. G. Kang S. M. Hussain A. Shahzad R. Seo C. W.. (2017). *Bacillus aryabhattai* SRB02 tolerates oxidative and nitrosative stress and promotes the growth of soybean by modulating the production of phytohormones. PLoS One 12:e0173203. doi: 10.1371/journal.pone.0173203, PMID: 28282395 PMC5345817

[ref68] Patil J. R. Mhatre K. J. Yadav K. Yadav L. S. Srivastava S. Nikalje G. C. (2024). Flavonoids in plant-environment interactions and stress responses. Discov. Plants 1, 1–19. doi: 10.1007/s44372-024-00063-6, PMID: 40881164

[ref69] Pavlović D. Nikolić B. Đurović S. Waisi H. Anđelković A. Marisavljević D.. (2014). Chlorophyll as a measure of plant health: agroecological aspects. Pestic. Fitomed. 29, 21–34. doi: 10.2298/pif1401021p

[ref70] Peng M. Wang D. Lui L. M. Nielsen T. Tian R. Kempher M. L.. (2022). Genomic features and pervasive negative selection in *Rhodanobacter* strains isolated from nitrate and heavy metal contaminated aquifer. Microbiol. Spectr. 10, e02591–e02521. doi: 10.1128/spectrum.02591-21, PMID: 35107332 PMC8809349

[ref71] Qin H. Wang Z. Sha W. Song S. Qin F. Zhang W. (2024). Role of plant-growth-promoting rhizobacteria in plant machinery for soil heavy metal detoxification. Microorganisms 12:700. doi: 10.3390/microorganisms12040700, PMID: 38674644 PMC11052264

[ref72] Qureshi M. A. Shahzad H. Saeed M. S. Ullah S. Ali M. A. Mujeeb F.. (2019). Relative potential of rhizobium species to enhance the growth and yield attributes of cotton (*Gossypium hirsutum* L.). Eurasian J. Soil Sci. 8, 159–166. doi: 10.18393/ejss.544747

[ref73] Rahim H. U. Ali W. Uddin M. Ahmad S. Khan K. Bibi H.. (2025). Abiotic stresses in soils, their effects on plants, and mitigation strategies: a literature review. Chem. Ecol. 41, 552–585. doi: 10.1080/02757540.2024.2439830

[ref74] Samsami H. Maali-Amiri R. (2024). Global insights into intermediate metabolites: signaling, metabolic divergence and stress response modulation in plants. Plant Physiol. Biochem. 213:108862. doi: 10.1016/j.plaphy.2024.108862, PMID: 38917735

[ref9005] Sanches Silva A. Reboredo-Rodríguez P. Sanchez-Machado D. I. López-Cervantes J. Barreca D. Pittala V.. (2020). Evaluation of the status quo of polyphenols analysis: Part II—Analysis methods and food processing effects[J]. Compr. Rev. Food. Sci. Food. Saf. 19, 3219–3240. doi: 10.1111/1541-4337.12626 33337047

[ref75] Shi Z. Guo X. Lei Z. Wang Y. Yang Z. Niu J.. (2023). Screening of high-efficiency nitrogen-fixing bacteria from the traditional Chinese medicine plant *Astragalus mongolicus* and its effect on plant growth promotion and bacterial communities in the rhizosphere. BMC Microbiol. 23:292. doi: 10.1186/s12866-023-03026-1, PMID: 37845638 PMC10578054

[ref76] Shi G. Hu J. Ding F. Li S. Shi W. Chen Y. (2022). Exogenous *Pseudomonas aeruginosa* application improved the phytoremediation efficiency of *Lolium multiflorum lam* on cu–cd co-contaminated soil. Environ. Technol. Innov. 27:102489. doi: 10.1016/j.eti.2022.102489

[ref77] Solomon W. Janda T. Molnár Z. (2024). Unveiling the significance of rhizosphere: implications for plant growth, stress response, and sustainable agriculture. Plant Physiol. Biochem. 206:108290. doi: 10.1016/j.plaphy.2023.108290, PMID: 38150841

[ref78] Song J. Cao X. An R. Ding H. Wang W. Zhou Y.. (2025). Physiological adaptation to different heavy metal stress in seedlings of halophyte *Suaeda liaotungensis*. Biology 14:260. doi: 10.3390/biology14030260, PMID: 40136518 PMC11940190

[ref79] Sultana R. Jashim A. I. I. Islam S. M. N. Rahman M. H. Haque M. M. (2024). Bacterial endophyte *Pseudomonas mosselii* PR5 improves growth, nutrient accumulation, and yield of rice (*Oryza sativa* L.) through various application methods. BMC Plant Biol. 24:1030. doi: 10.1186/s12870-024-05649-6, PMID: 39478459 PMC11523849

[ref80] Sun H. Gao P. Dong J. Zhao Q. Xue P. Geng L.. (2023). Rhizosphere bacteria regulated arsenic bioavailability and accumulation in the soil–Chinese cabbage system. Ecotoxicol. Environ. Saf. 249:114420. doi: 10.1016/j.ecoenv.2022.114420, PMID: 36521270

[ref9006] Talwar H. K. Chatli A. S. (2020). Search for the Holy Grail: Rhizobium[J]. Ind. J. Pure App. Biosci. 8, 98–114. doi: 10.18782/2582-2845.8256

[ref81] Tang H. Meng G. Xiang J. Mahmood A. Xiang G. SanaUllah. (2022). Toxic effects of antimony in plants: reasons and remediation possibilities—A review and future prospects. Front. Plant Sci. 13:1011945. doi: 10.3389/fpls.2022.1011945, PMID: 36388491 PMC9643749

[ref82] Tian Z. Li G. Tang W. Zhu Q. Li X. Du C.. (2022). Role of *Sedum alfredii* and soil microbes in the remediation of ultra-high content heavy metals contaminated soil. Agric. Ecosyst. Environ. 339:108090. doi: 10.1016/j.agee.2022.108090

[ref83] Ullah I. Anwar Y. Siddiqui M. F. Alsulami N. Ullah R. (2024). Phytoremediation of arsenic (as) in rice plants, mediated by *Bacillus subtilis* strain IU31 through antioxidant responses and phytohormones synthesis. Environ. Pollut. 355:124207. doi: 10.1016/j.envpol.2024.124207, PMID: 38795816

[ref84] Vidya C. S. N. Shetty R. Vaculíková M. Vaculík M. (2022). Antimony toxicity in soils and plants, and mechanisms of its alleviation. Environ. Exp. Bot. 202:104996. doi: 10.1016/j.envexpbot.2022.104996

[ref85] Wang X. Luo S. Chen Y. Zhang R. Lei L. Lin K.. (2023). Potential of *Miscanthus floridulus* associated with endophytic bacterium *Bacillus cereus* BL4 to remediate cadmium contaminated soil. Sci. Total Environ. 857:159384. doi: 10.1016/j.scitotenv.2022.159384, PMID: 36240921

[ref86] Wang J. Wang C. Wu X. Zhang J. Zhao G. Hou Y.. (2023). Effects of moderate drought extension on bacterial network structure in the rhizosphere soil of Leymus chinensis in semi-arid grasslands. Front. Microbiol. 14:1217557. doi: 10.3389/fmicb.2023.1217557, PMID: 37637130 PMC10448527

[ref87] Wang Q. Wang J. Zhang Z. Li M. Wang D. Zhang P.. (2024). Microbial metabolic traits drive the differential contribution of microbial necromass to soil organic carbon between the rhizosphere of absorptive roots and transport roots. Soil Biol. Biochem. 197:109529. doi: 10.1016/j.soilbio.2024.109529

[ref8001] Wani P. A. Khan M. S. Zaidi A. (2008). Effect of metal-tolerant plant growth-promoting Rhizobium on the performance of pea grown in metal-amended soil[J]. Arch. Environ. Contam. Toxicol. 55, 33–42. doi: 10.1007/s00244-007-9097-y 18166984

[ref89] Wu Y. Wang H. Peng L. Zhao H. Zhang Q. Tao Q.. (2024). Root-soil-microbiome interaction in the rhizosphere of Masson pine (*Pinus massoniana*) under different levels of heavy metal pollution. Ecotoxicol. Environ. Saf. 283:116779. doi: 10.1016/j.ecoenv.2024.116779, PMID: 39083870

[ref90] Wu Y. Zhao H. Xiao M. Liu H. He H. Peng L.. (2025). A plant growth-promoting bacterium supports cadmium detoxification of rice by inducing phenylpropanoid and flavonoid biosynthesis. J. Hazard. Mater. 484:136795. doi: 10.1016/j.jhazmat.2024.136795, PMID: 39647335

[ref91] Xing W. Gai X. Xue L. Li S. Zhang X. Ju F.. (2024). Enriched rhizospheric functional microbiome may enhance adaptability of *Artemisia lavandulaefolia* and *Betula luminifera* in antimony mining areas. Front. Microbiol. 15:1348054. doi: 10.3389/fmicb.2024.1348054, PMID: 38577689 PMC10993014

[ref93] Yan Z. Sun X. Xu Y. Zhang Q. Li X. (2017). Accumulation and tolerance of mangroves to heavy metals: a review. Curr. Pollut. Rep. 3, 302–317. doi: 10.1007/s40726-017-0066-4

[ref94] Ye L. Qiu S. Li X. Jiang Y. Jing C. (2018). Antimony exposure and speciation in human biomarkers near an active mining area in Hunan, China. Sci. Total Environ. 640, 1–8. doi: 10.1016/j.scitotenv.2018.05.267, PMID: 29852442

[ref95] Zainab N. Amna Khan A. A. Azeem M. A. Ali B. Wang T.. (2021). PGPR-mediated plant growth attributes and metal extraction ability of *Sesbania sesban* L. in industrially contaminated soils. Agronomy 11:1820. doi: 10.3390/agronomy11091820

[ref96] Zhang N. Jiang H. Zhou Z. Wang Y. Qi D. Zhou S.. (2025). Response of the nosZ-type denitrifying microbial community and metabolic characteristics to precipitation changes in the alpine wetland. Front. Microbiol. 16:1581432. doi: 10.3389/fmicb.2025.1581432, PMID: 40356652 PMC12067595

[ref97] Zhang J. Liu S. Liu C. B. Zhang M. Fu X. Q. Wang Y. L.. (2023). Natural variants of molybdate transporters contribute to yield traits of soybean by affecting auxin synthesis. Curr. Biol. 33, 5355–5367.e5. doi: 10.1016/j.cub.2023.10.072, PMID: 37995699

[ref98] Zhang Y. Wang J. Zhang X. Qin W. Si Y. (2025). Enhanced generation of reactive oxygen species by iron minerals for Sb (III) photooxidation: the vital role of Fe (II). J. Environ. Chem. Eng. 13:118504. doi: 10.1016/j.jece.2025.118504

[ref99] Zhang H. Xu Z. Guo K. Huo Y. He G. Sun H.. (2020). Toxic effects of heavy metal cd and Zn on chlorophyll, carotenoid metabolism and photosynthetic function in tobacco leaves revealed by physiological and proteomics analysis. Ecotoxicol. Environ. Saf. 202:110856. doi: 10.1016/j.ecoenv.2020.110856, PMID: 32629202

[ref100] Zhao S. Shi T. Terada A. Riya S. (2023). Evaluation of pollution level, spatial distribution, and ecological effects of antimony in soils of mining areas: A review. Int. J. Environ. Res. Public Health 20:242. doi: 10.3390/ijerph20010242, PMID: 36612564 PMC9819699

[ref101] Zhao Q. Zhang H. Zhang Z. Chen Z. Han H. (2024). Exogenous inoculation with heavy metal-immobilizing bacteria roused the key rhizosphere bacterial community and metabolites of wheat to inhibit cadmium absorption. J. Environ. Chem. Eng. 12:113631. doi: 10.1016/j.jece.2024.113631

[ref102] Zheng Y. Tang J. Liu C. Liu X. Luo Z. Zou D.. (2023). Alleviation of metal stress in rape seedlings (*Brassica napus* L.) using the antimony-resistant plant growth-promoting rhizobacteria *Cupriavidus* sp. S-8-2. Sci. Total Environ. 858:159955. doi: 10.1016/j.scitotenv.2022.159955, PMID: 36372176

[ref103] Zhou L. Liu W. (2024). Pollution of four heavy metal elements in dried chili peppers in Guizhou Province and its health risk assessment. Sci. Rep. 14:17759. doi: 10.1038/s41598-024-68564-8, PMID: 39085336 PMC11291500

[ref104] Zhou L. Ye B. Xia S. (2019). Structural characteristics of cake layer in membrane bioreactor with chromate exposure. Ecotoxicol. Environ. Saf. 169, 583–589. doi: 10.1016/j.ecoenv.2018.11.056, PMID: 30476820

[ref105] Zhou X. Zhang X. Ma C. Wu F. Jin X. Dini-Andreote F.. (2022). Biochar amendment reduces cadmium uptake by stimulating cadmium-resistant PGPR in tomato rhizosphere. Chemosphere 307:136138. doi: 10.1016/j.chemosphere.2022.136138, PMID: 36002065

[ref106] Zhu J. Li M. Whelan M. (2018). Phosphorus activators contribute to legacy phosphorus availability in agricultural soils: A review. Sci. Total Environ. 612, 522–537. doi: 10.1016/j.scitotenv.2017.08.095, PMID: 28865270

[ref107] Zhu Y. Yang J. Zhang J. Tong Y. Su H. Rensing C.. (2025). Assessment of ecological recovery potential of various plants in soil contaminated by multiple metal (loid) s at various sites near XiKuangShan mine. Land 14:223. doi: 10.3390/land14020223

