# Supplementary material for: Antimony-resistant PGPR mitigates Sb toxicity and accumulation in peppers by restructuring rhizosphere microorganisms
Source: Front Microbiol. 2025 Oct 1;16:1658223. doi: 10.3389/fmicb.2025.1658223 (PMC12521277; doi:10.3389/fmicb.2025.1658223)
Supplement: Supplementary file 1 [file Data_Sheet_1.docx]

**Supporting information**

**Antimony-Resistant Plant Growth-Promoting Rhizobacterium *Cupriavidus* sp. S- 8-2 Reduces Sb Toxicity and Accumulation in Pepper Plants ( *Capsicum annuum* L.) by Restructuring Rhizosphere Microbiota**

**Xiangquan Shenga,†, Jianzhong Zhua,†, Wenqian Lia, Juan Wan a, Kangbo Wu a,**

**Pan Yanga, Renyang Duana, Zeliang Yanga, Jing Baia,*,†, Yu Zhenga,b,*,†**

^a^*Hunan University of Humanities, Science and Technology, Loudi, Hunan 417000,*

*China*

^b^*Hunan Provincial Collaborative Innovation Center for Field Weeds Control, Hunan University of Humanities, Science and Technology, Loudi, Hunan 417000, China*

* **Corresponding author**:

E-mail: [zhengyu7175@163.com](mailto:zhengyu7175@163.com)

^†^These authors contributed equally to this work.

**List of supporting information**

**Supplementary Methods**

# S1: Determination of total flavonoid content

Specifically, 50 µl of the above methanolic extract was mixed with 150 µL of 5% sodium nitrite (NaNO₂) solution and incubated at room temperature for 5 minutes. Then, 150 μL 10% AlCl₃ and 1,000 μL 1 M sodium acetate were added sequentially. The final volume was adjusted to 5 mL with distilled water. After an additional 6-min incubation,

the absorbance of the resulting solution was measured at 380 nm using a spectrophotometer. A standard calibration curve was constructed using rutin solutions with concentrations ranging from 0 to 100 μg/mL. The total flavonoid content was expressed as rutin equivalents (RE) per gram of dry weight (mg RE/g DW), calculated according to the linear regression equation derived from the standard curve.

# S2: DNA sequencing and data process

For bacterial community analysis, a fragment of the 16S rRNA gene encompassing the V3 and V4 regions was amplified using the primer pair 341F (5′- CCTAYGGGRBGCASCAG-3′) and 806R (5′-GGACTACNNGGGTATCTAAT-3′)

(Fietz et al., 2018).

Alpha diversity indices, including the ACE, Chao1, Shannon, and Simpson indices, as well as beta diversity, were calculated using QIIME2. Differences in alpha diversity among samples were analyzed using t-tests. Venn diagrams were generated to visualize shared and unique ASVs across different samples. Principal coordinate analysis (PCoA) and ANOSIM, which are based on Bray-Curtis dissimilarity, were performed to visually compare the bacterial and fungal community structures. Linear discriminant analysis (LDA) effect size (LEfSe) (LDA score >4) was used to identify significantly different taxa between samples. The functional prediction of the microbial communities was conducted via PICRUSt2 (Yang et al., 2023). The selected predictive functional categories from KEGG levels 1 and 2 were analyzed in detail.

# S3: network analysis

Network construction was performed via the "Hmisc" and "igraph" R packages, applying Spearman’s correlation coefficient. Only strong and significant correlations (r > |0.6|; p < 0.05) were included in the analysis. Gephi (version v 0.10.1) was employed for network visualization, while network attributes such as modularity, average degree, density, and the number of nodes and edges were quantified as reported by Imparato et al., (2024). Operational taxonomic units (Outs) were represented as nodes, with node size reflecting the number of connections (degree), whereas node color indicated microbial taxa. Edges depict either positive or negative correlations between nodes.

Redundancy analysis (RDA) was conducted to evaluate the relationships between the soil factors and the compositions of the bacterial and fungal communities at the phylum level. The determination of P values was based on 999 Monte Carlo permutations. The Mantel test, with 999 permutations, was performed via the "vegan" and "ggplot2" R packages to evaluate the associations between rhizosphere environmental parameters and microbial communities based on Pearson correlation (p

< 0.05). Spearman correlations were used to determine the relationships of rhizosphere environmental parameters with microbial phyla and genera.

**Table S1.** Overview of sequencing number before and after each step of analyses.

| Sample ID | Input | Filtered | Denoised | Merged | Non#chimeric | Non#singleton |
| --- | --- | --- | --- | --- | --- | --- |
| UCK1 | 88,172 | 81,023 | 78,006 | 61,028 | 49,215 | 48,900 |
| UCK2 | 86,390 | 79,363 | 77,233 | 66,234 | 60,552 | 60,397 |
| UCK3 | 83,181 | 76,862 | 74,390 | 62,221 | 55,067 | 54,840 |
| UCK4 | 80,506 | 74,168 | 71,357 | 56,172 | 45,016 | 44,710 |
| ICK1 | 87,563 | 80,808 | 77,187 | 58,452 | 47,084 | 46,670 |
| ICK2 | 81,807 | 75,234 | 72,061 | 55,840 | 46,909 | 46,606 |
| ICK3 | 85,297 | 78,529 | 75,130 | 58,460 | 48,858 | 48,489 |
| ICK4 | 85,752 | 79,252 | 76,235 | 59,866 | 50,788 | 50,449 |
| ULT1 | 82,986 | 76,931 | 73,645 | 57,577 | 46,365 | 45,968 |
| ULT2 | 82,427 | 76,601 | 73,352 | 57,364 | 47,596 | 47,249 |

| ULT3 | 81,216 | 75,590 | 71,975 | 54,747 | 43,404 | 43,041 |
| --- | --- | --- | --- | --- | --- | --- |
| ULT4 | 82,443 | 76,705 | 73,109 | 55,424 | 42,817 | 42,413 |
| ILT1 | 86,773 | 79,967 | 77,065 | 60,888 | 48,755 | 48,458 |
| ILT2 | 80,142 | 74,521 | 71,786 | 58,679 | 49,975 | 49,710 |
| ILT3 | 81,533 | 75,421 | 72,756 | 59,133 | 50,622 | 50,342 |
| ILT4 | 82,223 | 76,257 | 73,665 | 59,536 | 49,615 | 49,345 |
| UHT1 | 87,992 | 81,995 | 78,934 | 62,903 | 50,415 | 50,065 |
| UHT2 | 84,509 | 78,678 | 75,817 | 59,177 | 47,592 | 47,258 |
| UHT3 | 85,704 | 79,455 | 76,390 | 60,286 | 48,522 | 48,132 |
| UHT4 | 80,039 | 74,086 | 71,079 | 56,463 | 46,339 | 45,998 |
| IHT1 | 83,517 | 69,246 | 66,771 | 54,571 | 45,752 | 45,449 |
| IHT2 | 80,870 | 74,805 | 71,821 | 57,933 | 47,145 | 46,837 |
| IHT3 | 80,566 | 74,915 | 72,123 | 58,937 | 48,712 | 48,405 |

| IHT4 | 81,802 | 75,587 | 72,048 | 54,899 | 41,249 | 40,826 |
| --- | --- | --- | --- | --- | --- | --- |

Note: UCK, ICK, ULT, ILT, UHT, and IHT respectively represented six treatments: uninoculated control (0 mg/kg Sb; UCK), inoculated control (0 mg/kg Sb + *Cupriavidus* sp. S-8-2; ICK), uninoculated low Sb stress (500 mg/kg Sb; ULH), inoculated low Sb stress (500 mg/kg Sb

+ *Cupriavidus* sp. S-8-2; ILH), uninoculated high Sb stress (1000 mg/kg Sb; UHT), and inoculated high Sb stress (1000 mg/kg Sb + *Cupriavidus* sp. S-8-2; IHT).

**Table S2.** The top 30 differentially abundant phyla in the rhizosphere soil of capsicum.

| Taxonomy | UCK | ICK | ULT | ILT | UHT | IHT |
| --- | --- | --- | --- | --- | --- | --- |
| Proteobacteria | 0.399178 | 0.429190 | 0.431480 | 0.446158 | 0.463286 | 0.520995 |
| Actinobacteria | 0.283303 | 0.257889 | 0.262821 | 0.279912 | 0.255465 | 0.225931 |
| Chloroflexi | 0.097600 | 0.096378 | 0.093825 | 0.078144 | 0.069695 | 0.054254 |
| Gemmatimonadetes | 0.074561 | 0.075560 | 0.074694 | 0.053737 | 0.067750 | 0.062404 |
| Bacteroidetes | 0.037685 | 0.039868 | 0.050378 | 0.055466 | 0.060536 | 0.056304 |

| Planctomycetes | 0.040568 | 0.033002 | 0.033624 | 0.032761 | 0.036203 | 0.035315 |
| --- | --- | --- | --- | --- | --- | --- |
| Acidobacteria | 0.034535 | 0.035326 | 0.026117 | 0.020623 | 0.019975 | 0.015083 |
| Firmicutes | 0.009839 | 0.007808 | 0.007181 | 0.006976 | 0.005846 | 0.007463 |
| Patescibacteria | 0.007609 | 0.007616 | 0.006730 | 0.006473 | 0.006853 | 0.005414 |
| Cyanobacteria | 0.005335 | 0.004209 | 0.003618 | 0.010576 | 0.003711 | 0.006937 |
| Armatimonadetes | 0.002664 | 0.002866 | 0.001663 | 0.001884 | 0.002700 | 0.002464 |
| Verrucomicrobia | 0.001600 | 0.001794 | 0.003420 | 0.000927 | 0.001778 | 0.001624 |
| BRC1 | 0.000742 | 0.002230 | 0.000461 | 0.002654 | 0.001970 | 0.001330 |
| FBP | 0.000978 | 0.001671 | 0.001462 | 0.001767 | 0.001198 | 0.001637 |
| Euryarchaeota | 0.000515 | 0.000706 | 0.000455 | 0.000451 | 0.000646 | 0.000791 |
| Dependentiae | 0.000706 | 0.000577 | 0.000287 | 0.000201 | 0.000617 | 0.001010 |
| Nitrospirae | 0.000752 | 0.001053 | 0.000428 | 0.000400 | 0.000372 | 0.000250 |
| WPS-2 | 0.000641 | 0.000592 | 0.000150 | 0.000447 | 0.000392 | 0.000310 |

| Thaumarchaeota | 0.000106 | 0.000250 | 0.000122 | 6.52E-05 | 6.26E-05 | 9.60E-05 |
| --- | --- | --- | --- | --- | --- | --- |
| Chlamydiae | 9.30E-05 | 0.000149 | 0.000158 | 0 | 0.00011148 | 0 |
| Fibrobacteres | 2.51E-05 | 0.000140 | 6.21E-05 | 1.55E-05 | 1.50E-05 | 0 |
| WS4 | 2.05E-05 | 3.55E-05 | 4.65E-05 | 0 | 6.86E-05 | 4.22E-05 |
| Elusimicrobia | 6.85E-05 | 5.84E-05 | 0 | 1.01E-05 | 0 | 0 |
| Halanaerobiaeota | 0 | 1.03E-05 | 3.92E-05 | 6.53E-05 | 1.56E-05 | 0 |
| Deinococcus-Thermus | 0 | 0 | 3.88E-05 | 1.51E-05 | 5.71E-05 | 1.03E-05 |
| Hydrogenedentes | 1.24E-05 | 0 | 5.17E-05 | 0 | 1.09E-05 | 0 |
| Crenarchaeota | 1.37E-05 | 1.07E-05 | 1.63E-05 | 0 | 0 | 0 |
| Zixibacteria | 0 | 1.61E-05 | 1.06E-05 | 0 | 0 | 0 |
| Omnitrophicaeota | 0 | 0 | 0 | 0 | 0 | 1.07E-05 |
| Schekmanbacteria | 9.12E-06 | 0 | 0 | 0 | 0 | 0 |

Note: UCK, ICK, ULT, ILT, UHT, and IHT respectively represented six treatments: uninoculated control (0 mg/kg Sb; UCK), inoculated control

(0 mg/kg Sb + *Cupriavidus* sp. S-8-2; ICK), uninoculated low Sb stress (500 mg/kg Sb; ULH), inoculated low Sb stress (500 mg/kg Sb

+ *Cupriavidus* sp. S-8-2; ILH), uninoculated high Sb stress (1000 mg/kg Sb; UHT), and inoculated high Sb stress (1000 mg/kg Sb + *Cupriavidus* sp. S-8-2; IHT).

**Table S3.** The top 30 differentially abundant genera at the genus level in the rhizosphere soil of capsicum.

| Taxonomy | UCK | ICK | ULT | ILT | UHT | IHT |
| --- | --- | --- | --- | --- | --- | --- |
| *Rhodanobacter* | 0.078882 | 0.063975 | 0.079816 | 0.062960 | 0.073229 | 0.068323 |
| *JG30-KF-AS9* | 0.060763 | 0.055273 | 0.061886 | 0.047149 | 0.043053 | 0.033902 |
| *Pseudolabrys* | 0.025879 | 0.024953 | 0.030695 | 0.033100 | 0.036048 | 0.031724 |
| *Chujaibacter* | 0.018764 | 0.015747 | 0.024754 | 0.028157 | 0.024364 | 0.023292 |
| *Nocardioides* | 0.017502 | 0.019289 | 0.017999 | 0.019515 | 0.017822 | 0.015515 |
| *SC-I-84* | 0.022083 | 0.023190 | 0.016616 | 0.016664 | 0.014180 | 0.009471 |
| *Pseudaminobacter* | 0.009526 | 0.011313 | 0.016217 | 0.019745 | 0.014657 | 0.017978 |

| *Jatrophihabitans* | 0.012984 | 0.018626 | 0.016138 | 0.018661 | 0.013902 | 0.017576 |
| --- | --- | --- | --- | --- | --- | --- |
| *Nitrolancea* | 0.012495 | 0.014922 | 0.012168 | 0.012460 | 0.010120 | 0.009679 |
| *Devosia* | 0.010795 | 0.009408 | 0.013715 | 0.012194 | 0.011253 | 0.009345 |
| *Lysobacter* | 0.011019 | 0.011585 | 0.005120 | 0.014441 | 0.011102 | 0.011435 |
| *Sphingobium* | 0.005248 | 0.011058 | 0.008820 | 0.012756 | 0.013306 | 0.016698 |
| *67-14* | 0.010583 | 0.008157 | 0.010974 | 0.010727 | 0.012041 | 0.009019 |
| *Luteimonas* | 0.001468 | 0.003203 | 0.008062 | 0.017348 | 0.011030 | 0.013634 |
| *Sphingomonas* | 0.006101 | 0.008499 | 0.004949 | 0.008340 | 0.010081 | 0.013063 |
| *Sphingopyxis* | 0.007449 | 0.009346 | 0.004541 | 0.008990 | 0.006836 | 0.013743 |
| *Aeromicrobium* | 0.011950 | 0.011237 | 0.004919 | 0.008607 | 0.004224 | 0.004830 |
| *Luteibacter* | 0.003703 | 0.003584 | 0.009155 | 0.005124 | 0.006770 | 0.015275 |
| *Porphyrobacter* | 0.006630 | 0.006459 | 0.006241 | 0.005854 | 0.008527 | 0.009095 |
| *Acidipila* | 0.008710 | 0.008740 | 0.007352 | 0.006661 | 0.005689 | 0.004173 |

| *Streptomyces* | 0.006350 | 0.005657 | 0.007692 | 0.006801 | 0.006648 | 0.006084 |
| --- | --- | --- | --- | --- | --- | --- |
| *Gemmatimonas* | 0.007251 | 0.006249 | 0.007235 | 0.005279 | 0.005793 | 0.005937 |
| *Burkholderia-Caballeronia-Paraburkhol* | *deria* 0.003333 | 0.003010 | 0.004991 | 0.007113 | 0.009614 | 0.007784 |
| *Subgroup_6* | 0.007384 | 0.007960 | 0.006303 | 0.004521 | 0.004587 | 0.004216 |
| *Novosphingobium* | 0.004292 | 0.005629 | 0.009681 | 0.003152 | 0.004939 | 0.005507 |
| *Mycobacterium* | 0.005354 | 0.005652 | 0.005056 | 0.004477 | 0.004995 | 0.006852 |
| *Marmoricola* | 0.006018 | 0.006024 | 0.005584 | 0.005787 | 0.004242 | 0.003578 |
| *Haliangium* | 0.004254 | 0.005572 | 0.005188 | 0.005585 | 0.005085 | 0.005231 |
| *Massilia* | 0.001267 | 0.002416 | 0.005162 | 0.003485 | 0.008505 | 0.009069 |
| *Allorhizobium-Neorhizobium-Pararhizob* | *ium-Rhizobium* 0.002348 | 0.003192 | 0.002761 | 0.008486 | 0.003346 | 0.008258 |
| *WD2101_soil_group* | 0.005105 | 0.004599 | 0.003575 | 0.004418 | 0.005689 | 0.004866 |
| *Taibaiella* | 0.005131 | 0.006784 | 0.005308 | 0.002058 | 0.003622 | 0.005325 |
| *Nitrosospira* | 0.001778 | 0.003337 | 0.004106 | 0.005673 | 0.005269 | 0.006355 |

| *C0119* | 0.003976 | 0.007173 | 0.003655 | 0.004132 | 0.005218 | 0.001918 |
| --- | --- | --- | --- | --- | --- | --- |
| *Saccharimonadales* | 0.005284 | 0.005767 | 0.003770 | 0.003840 | 0.004434 | 0.004719 |
| *Sericytochromatia* | 0.003806 | 0.003044 | 0.002140 | 0.008185 | 0.002303 | 0.005497 |
| *Conexibacter* | 0.006030 | 0.005824 | 0.004085 | 0.002184 | 0.003420 | 0.003858 |
| *Planctomicrobium* | 0.002738 | 0.001112 | 0.004908 | 0.005715 | 0.005579 | 0.003863 |
| *Micropepsis* | 0.006102 | 0.005412 | 0.004185 | 0.003502 | 0.002294 | 0.001382 |
| *Altererythrobacter* | 0.000504 | 0.002834 | 0.002550 | 0.003564 | 0.005603 | 0.007812 |
| *Ellin6067* | 0.005122 | 0.005013 | 0.002579 | 0.003268 | 0.003185 | 0.003286 |
| *Parvibaculum* | 0.000933 | 0.001248 | 0.003502 | 0.003054 | 0.007677 | 0.005791 |
| *Hyphomicrobium* | 0.003192 | 0.003650 | 0.003715 | 0.004214 | 0.003578 | 0.003803 |
| *OLB14* | 0.004139 | 0.004042 | 0.003706 | 0.004559 | 0.002609 | 0.002385 |
| *JG30-KF-CM45* | 0.004228 | 0.003764 | 0.003380 | 0.003962 | 0.003215 | 0.002400 |
| *Bryobacter* | 0.004366 | 0.005406 | 0.003532 | 0.002669 | 0.002436 | 0.001596 |

| *Granulicella* | 0.006196 | 0.004844 | 0.002966 | 0.002827 | 0.002115 | 0.001012 |
| --- | --- | --- | --- | --- | --- | --- |
| *Gemmata* | 0.004267 | 0.004202 | 0.003528 | 0.002048 | 0.002706 | 0.002640 |
| *Bradyrhizobium* | 0.003126 | 0.002713 | 0.002946 | 0.003648 | 0.003451 | 0.003495 |
| *Acidibacter* | 0.004454 | 0.003766 | 0.003372 | 0.002568 | 0.002265 | 0.002369 |

Note: UCK, ICK, ULT, ILT, UHT, and IHT respectively represented six treatments: uninoculated control (0 mg/kg Sb; UCK), inoculated control (0 mg/kg Sb + *Cupriavidus* sp. S-8-2; ICK), uninoculated low Sb stress (500 mg/kg Sb; ULH), inoculated low Sb stress (500 mg/kg Sb

+ *Cupriavidus* sp. S-8-2; ILH), uninoculated high Sb stress (1000 mg/kg Sb; UHT), and inoculated high Sb stress (1000 mg/kg Sb + *Cupriavidus* sp. S-8-2; IHT).

**2. Supplementary Figures**

**
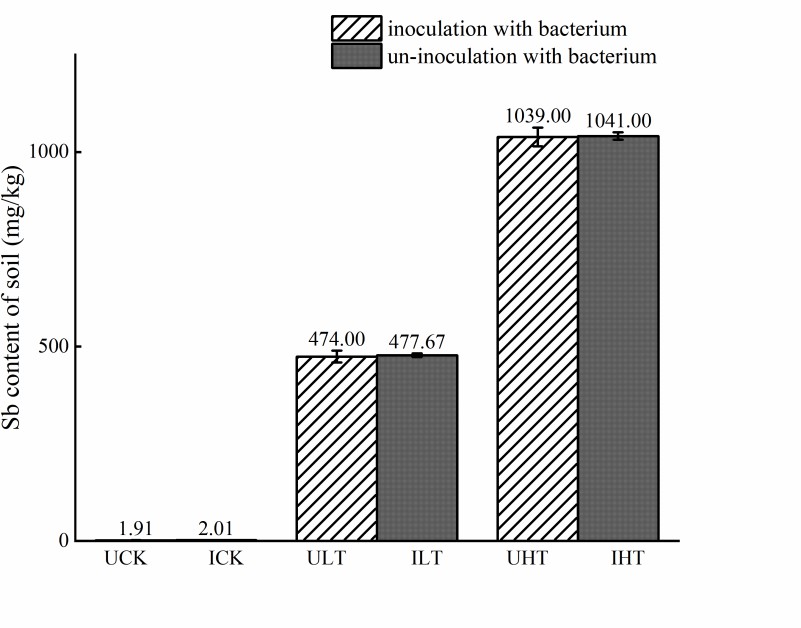
**

**Fig. S1.** Sb concentration validation. Data are presented as mean ± SE (N = 4). UCK, ICK, ULT, ILT, UHT, and IHT respectively represented six treatments: uninoculated control (0 mg/kg Sb; UCK), inoculated control (0 mg/kg Sb + *Cupriavidus* sp. S-8-2; ICK), uninoculated low Sb stress (500 mg/kg Sb; ULH), inoculated low Sb stress (500 mg/kg Sb + *Cupriavidus* sp. S-8-2; ILH), uninoculated high Sb stress (1000 mg/kg Sb; UHT), and inoculated high Sb stress (1000 mg/kg Sb + *Cupriavidus* sp. S-8-2; IHT).


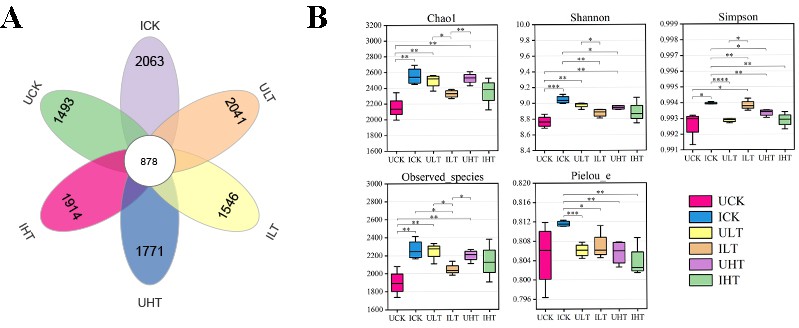


**Fig. S2.** Changes in the diversity of rhizosphere microflora under different treatments.

(A) Venn diagram depicting common and unique OUTs in different treatments. (B ) Boxplots representing changes in Chao1, Shannon, Simpson, Observed species and Pielou_e values under different treatments. Asterisks indicate significant differences between inoculated and uninoculated treatments within the same Sb level (**P* < 0.05,

***P* < 0.01, ****P* < 0.001; one-way ANOVA). Data are presented as mean ± SE (N = 4). UCK (0 mg/kg Sb group), ICK (0 mg/kg Sb + *Cupriavidus sp.* S-8-2 group), ULT(500mg/kg Sb group), ILT (500mg/kg Sb + *Cupriavidus* sp. S-8-2 group), UHT(1000 mg/kg Sb group) and IHT (1000 mg/kg Sb + *Cupriavidus* sp. S-8-2 group).


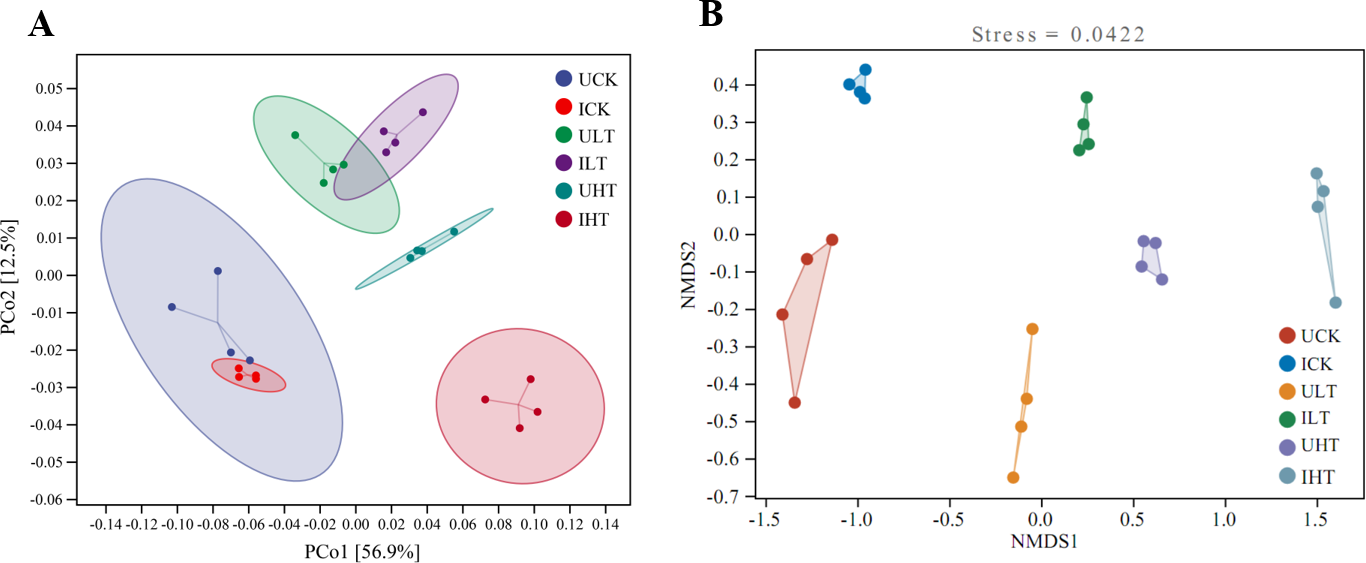


**Fig. S3.** Beta diversity analysis of rhizosphere microbial communities under Sb stress with or without *Cupriavidus* sp. S-8-2 inoculation. Principal Coordinates Analysis (PCoA) based on weighted UniFrac distances, showing the separation of microbial communities across treatments (A).Non-metric multidimensional scaling (NMDS) plot of microbial community composition. Each point represents a sample, colored by group. Point proximity approximates community dissimilarity (closer = more similar; farther


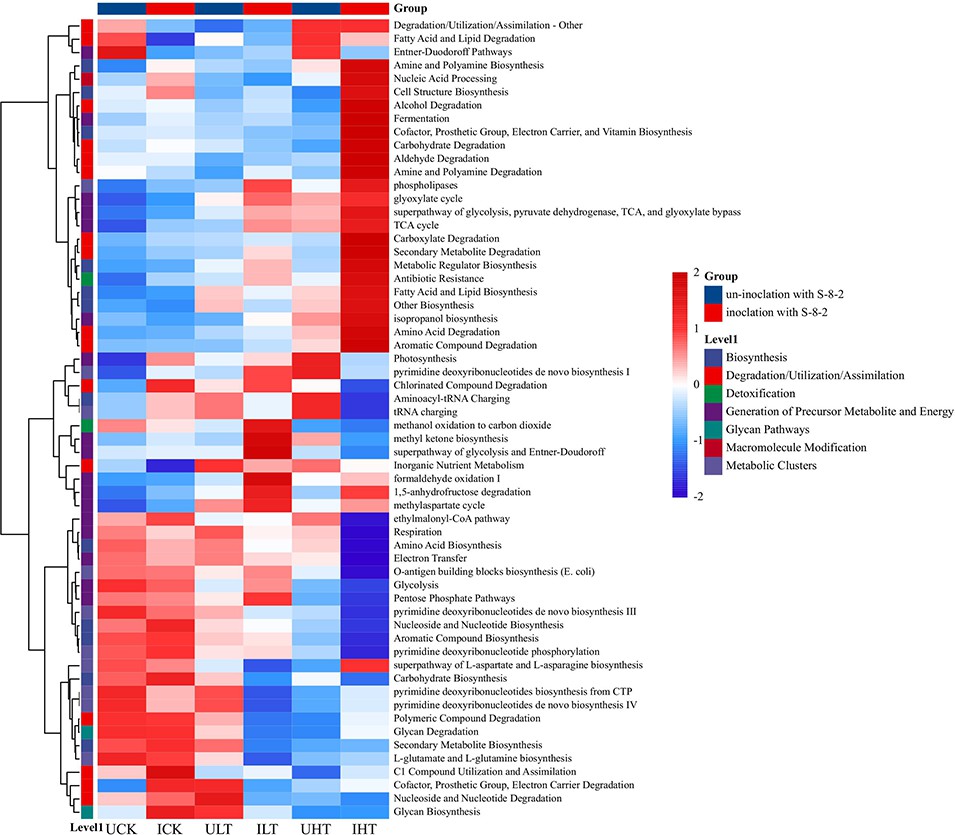
apart = more dissimilar). Elliptical dotted lines denote 95% confidence ellipses for groups with ≥4 samples (B). UCK (0 mg/kg Sb control), ICK (0 mg/kg Sb + *Cupriavidus* sp. S-8-2), ULT (500 mg/kg Sb), ILT (500 mg/kg Sb + *Cupriavidus* sp. S- 8-2), UHT (1000 mg/kg Sb), IHT (1000 mg/kg Sb + *Cupriavidus* sp. S-8-2).

**Fig. S4.** Metabolic pathway prediction via PICRUSt2. Stacked bars depict KEGG level- 1 and level-2 subcategories with differential abundance of microbial communities of the rhizosphere of pepper under Sb stress with or without *Cupriavidus* sp. S-8-2 inoculation. Data are presented as mean ± SE (N = 4). UCK (0 mg/kg Sb control), ICK (0 mg/kg Sb + *Cupriavidus* sp. S-8-2), ULT (500 mg/kg Sb), ILT (500 mg/kg Sb + *Cupriavidus* sp. S-8-2), UHT (1000 mg/kg Sb), IHT (1000 mg/kg Sb + *Cupriavidus* sp. S-8-2).

**Literature cited in supporting information**

Fietz, K., Rye Hintze, C.O., Skovrind, M., Kjærgaard Nielsen, T., Limborg, M.T., Krag, M.A., Palsbøll, P.J., Hestbjerg Hansen, L., Rask Møller, P., Gilbert, M.T.P., 2018. Mind the gut: genomic insights to population divergence and gut microbial composition of two marine keystone species. Microbiome 6, 82. <https://doi.org/10.1186/s40168-018-0467-7>

Imparato, D.O.; Cavalcante, J.V.F.; Dalmolin, R.J. easylayout: An R package for interactive force-directed layouts within RStudio. arXiv 2024, arXiv:2409.18280 https://doi.org/10.48550/arXiv.2409.18280

Yang, F., Jiang, H., Chang, G., Liang, S., Ma, K., Cai, Y., Tian, B., Shi, X., 2023. Effects of rhizosphere microbial communities on cucumber Fusarium wilt disease suppression. Microorganisms 11(6), 1576.

<https://doi.org/10.3390/microorganisms11061576>
